# Scalable in situ single-cell profiling by electrophoretic capture of mRNA using EEL FISH

**DOI:** 10.1038/s41587-022-01455-3

**Published:** 2022-09-22

**Authors:** Lars E. Borm, Alejandro Mossi Albiach, Camiel C. A. Mannens, Jokubas Janusauskas, Ceren Özgün, David Fernández-García, Rebecca Hodge, Francisca Castillo, Charlotte R. H. Hedin, Eduardo J. Villablanca, Per Uhlén, Ed S. Lein, Simone Codeluppi, Sten Linnarsson

**Affiliations:** 1 Division of Molecular Neurobiology, Department of Medical Biochemistry and Biophysics, Karolinska Institutet, Stockholm, Sweden; 2 Allen Institute for Brain Science, Seattle, WA USA; 3 Division of Immunology and Allergy, Department of Medicine, Karolinska Institutet and University Hospital, Stockholm, Sweden; 4 Department of Medicine, Solna Karolinska Institutet, Stockholm, Sweden; 5 Gastroenterology Unit, Patient Area Gastroenterology, Dermatovenereology, and Rheumatology, Karolinska University Hospital, Stockholm, Sweden; 6 Present Address: Rebus Biosystems, Santa Clara, CA USA

**Keywords:** Transcriptomics, Cellular neuroscience, Imaging

## Abstract

Methods to spatially profile the transcriptome are dominated by a trade-off between resolution and throughput. Here we develop a method named Enhanced ELectric Fluorescence in situ Hybridization (EEL FISH) that can rapidly process large tissue samples without compromising spatial resolution. By electrophoretically transferring RNA from a tissue section onto a capture surface, EEL speeds up data acquisition by reducing the amount of imaging needed, while ensuring that RNA molecules move straight down toward the surface, preserving single-cell resolution. We apply EEL on eight entire sagittal sections of the mouse brain and measure the expression patterns of up to 440 genes to reveal complex tissue organization. Moreover, EEL can be used to study challenging human samples by removing autofluorescent lipofuscin, enabling the spatial transcriptome of the human visual cortex to be visualized. We provide full hardware specifications, all protocols and complete software for instrument control, image processing, data analysis and visualization.

## Abstract (web summary)

EEL FISH profiles spatial transcriptomes in single cells in large, complex tissue samples.

## Main

The brain is a highly structured organ that comprises a vast diversity of cell types including many subtypes of excitatory and inhibitory neurons, astrocytes, oligodendrocytes and vascular and immune cells^1^. Cell numbers vary by cell type, from a few hundred cells (for example, hypothalamus *Pmch*^+^ neurons) to billions (for example, cerebellar granule neurons) and are organized in intricate spatial patterns on scales ranging from micrometers to centimeters. Studying the organization and spatial relationships between all these different cell types by microscopy is challenging because the diversity vastly outnumbers the handful of colors that can be simultaneously resolved. However, recent advances in multiplexing have made it possible to elucidate the complex structure of the brain in situ at higher resolution in health and disease^2–6^.

These newer spatial methods use either sequencing or microscopy to detect mRNA in space^7,8^. Sequencing-based methods transfer the mRNA of a tissue section onto a surface where transcripts are hybridized or ligated to spots of spatially barcoded oligonucleotides, which are then collected and resolved by sequencing^9–15^. These methods can measure the entire transcriptome using existing sequencing pipelines and can scale to large areas and high throughput by parallel sample processing. Nevertheless, they are limited by low capture efficiency, gaps between capture spots and high sequencing costs. Furthermore, spot-based spatial data are difficult to segment into true single-cell data and are commonly analyzed in terms of spots, not cells.

Microscopy-based methods rely on single-molecule fluorescence in situ hybridization (smFISH) or in situ sequencing to directly visualize RNA transcripts in tissue^2,5,6,16–21^. To be able to map many transcripts and overcome the limited color space resolvable by fluorescence microscopes, some smFISH methods use a barcoded approach in which different gene transcripts are encoded by a binary barcode that is detected by multiple cycles of labeling, imaging and stripping. Barcoding enables the detection of as many as 10,000 different RNA species^4,22^. Nevertheless, the analysis of large samples is time-consuming because of repeated imaging at high magnification using high-numerical aperture (NA) microscope objectives. The small field of view (FOV) and shallow depth of field of such objectives necessitate the imaging of many *xy* tiles and a large *z*-stack such that surveying only a few square millimeters can take days^3^ to weeks^2^.

One way to increase the imaging speed is to make the signal detectable by low-power microscope objectives characterized by a large FOV through signal amplification but sacrificing quantitative sensitivity^5,6,21^. However, the reduced resolution of low-magnification objectives causes an increase in optical crowding, in which signals from multiple RNA molecules cannot be separated, therefore preventing high multiplexing (Supplementary Fig. 1a).

Here we describe Enhanced ELectric FISH (EEL FISH), an smFISH-based method that combines high multiplexing with large-area imaging at high resolution. By electrophoretically transferring RNA onto a glass surface and removing the tissue, time-consuming imaging in the *z* axis can be minimized, while it also reduces background, speeds up RNA labeling and facilitates multiplexing by barcoding. Here we implement barcodes for up to 448 genes per color channel and demonstrate two-color imaging. Furthermore, electrophoresis reduced lateral dispersion of the captured RNA, when compared to RNA transfer by passive diffusion (as used by, for example, Visium^9^), resulting in a more faithful RNA blot that retains cellular resolution.

We applied EEL to a full sagittal section of the mouse brain where 440 genes were measured in a little over two days of imaging, which enabled the study of spatial regions, gradients and borders defined by gene expression. Moreover, the cells could be segmented to yield the spatial transcriptome profiles of more than 128,000 single cells, enabling clustering and visualization of cell-type-specific expression profiles in their spatial context. To further demonstrate the robustness, scalability and versatility of EEL, we then created a transcriptome atlas of the mouse brain comprising seven additional sagittal sections, where we examined spatial organization. Finally, we applied EEL to the human visual cortex and found that EEL greatly reduced the highly autofluorescent lipofuscin deposits that normally restrict RNA detection by smFISH in human tissue.

To aid implementation of EEL in other labs, we provide a detailed description of the hardware—schematics, parts list, build instructions—together with all the necessary software for instrument control, image processing, data analysis and visualization.

## Results

### EEL protocol

**Fig. 1:**
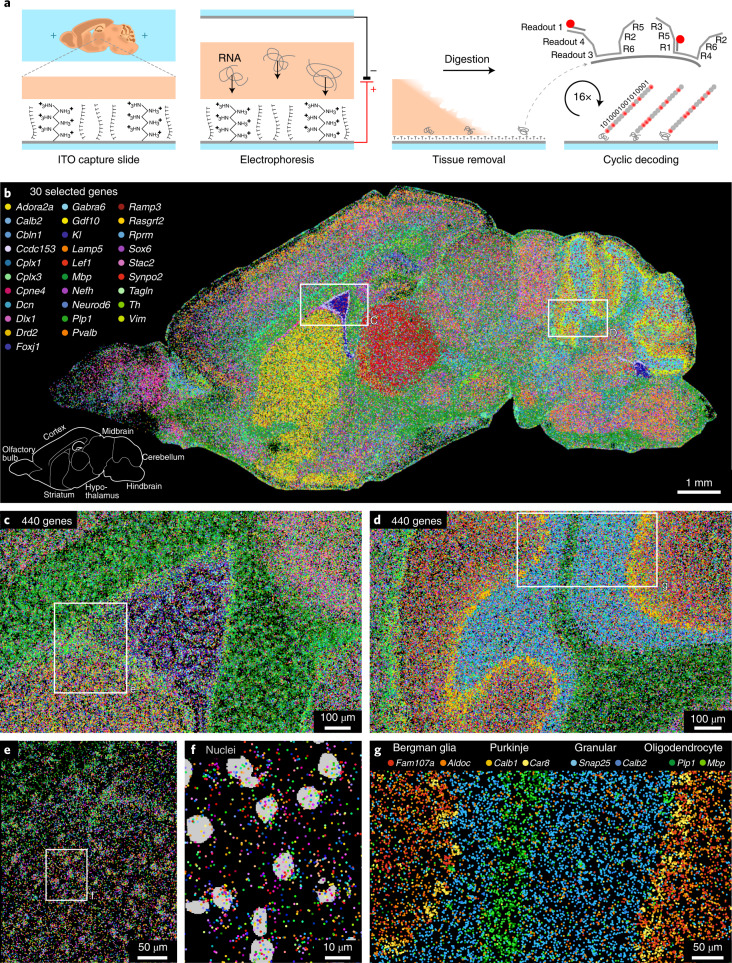
EEL method and 440-gene mouse dataset. **a**, Schematic illustration of the EEL protocol, including RNA transfer by electrophoresis, capture on the ITO slide, tissue removal and cyclic fluorescent decoding. **b**, Results of EEL on a sagittal mouse brain section, where every dot is a single molecule of RNA belonging to one of the 30 selected genes to highlight large anatomical structures. The total experiment contained 440 genes. **c**,**d**, Zoomed-in results for all genes around the lateral ventricle (**c**) and cerebellum (**d**). **e**–**g**, High-resolution imaging (<500 nm) yields subcellular resolution (**e**,**f**) and cell type marker expression patterns for small anatomical features (**g**).

We developed and optimized a protocol to transfer RNA from a tissue section onto a capture slide with high efficiency and minimal spatial distortion, by actively forcing the RNA onto the surface through electrophoresis. Briefly (see Methods for a detailed protocol), the RNA capture slide is a glass coverslip coated with an optically transparent and electrically conductive layer of indium tin oxide (ITO), which is modified with oligo(dT) and positively charged poly(d-lysine) to capture RNA both by hybridization and by electrostatic attraction (Fig. 1a). A 10-µm cryosection is placed onto the capture slide, and stained nuclei are imaged for cell segmentation. The tissue is permeabilized and an electric potential difference of 10 V cm^−1^ is applied for 20 min, where the capture slide acts as the anode (Supplementary Fig. 1b). When the RNA transfer has been completed, the tissue sample is digested, leaving only the captured RNA on the surface. Tissue removal speeds up the subsequent detection chemistry because reagents and probes do not need to diffuse through the tissue.

Next, to detect up to 448 species of RNA per color channel, we designed a set of binary codes with 6 positive bits of 16 bits total and a minimum of 4 bits of difference between any pair of codes^23^. For each desired target gene, a set of highly specific encoding probes was designed to tile the transcript, each carrying overhanging tails encoding the six positive bits of the selected barcode (Fig. 1a).

For each experiment, the full set of encoding probes for all target genes was pooled and hybridized upfront and was then detected by 16 cycles of fluorescent readout probe hybridization to the tails, imaging and Tris (2-carboxyethyl) phosphine (TCEP)-mediated fluorophore cleavage. The entire process was implemented on a custom-built open-source fluidic system, integrated with a commercial microscope to perform the barcode detection automatically (Fig. 1a and Supplementary Fig. 1c–e).

As proof of principle, we targeted 440 genes and eight empty-barcode controls, which after image processing and barcode decoding resulted in 8,871,209 detected RNA molecules with a false-positive rate of 0.025% ± 0.011% per gene. The spatial expression patterns could be analyzed both at the scale of the full tissue section (Fig. 1b), of smaller anatomical structures (Fig. 1c,d,g), and at the single-cell level (Fig. 1e,f).

### EEL transfer efficiency and sensitivity

**Fig. 2:**
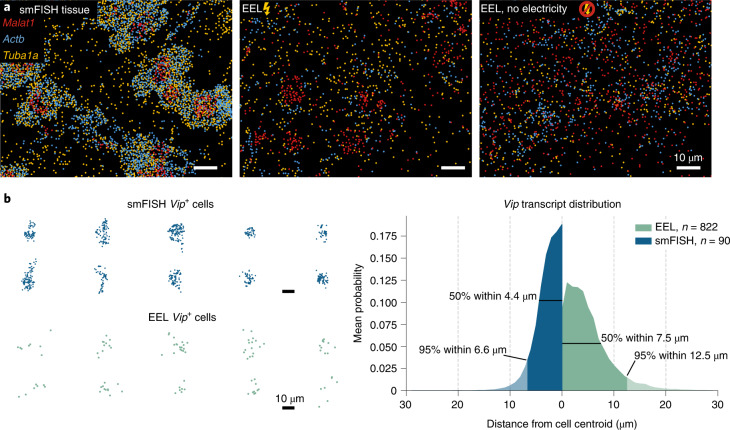
Electrophoresis enhances RNA blot accuracy. **a**, Three-gene smFISH in tissue (left), EEL with electrophoresis (middle) and EEL without electrophoresis (right). With electricity, the EEL RNA distribution looks more similar to the original tissue situation than without it. **b**, Distribution of *Vip* RNA in tissue smFISH (*n* = 90) and EEL (*n* = 822), indicating that samples processed with EEL show little lateral diffusion from the cell centroid.

We estimated the RNA transfer efficiency by comparing RNA numbers detected by smFISH directly after transfer and tissue digestion with those of a consecutive tissue section processed using regular in-tissue smFISH. We found that around 19% of the RNA was transferred onto the surface (Fig. 2a and Supplementary Fig. 2a–c). However, barcode detection and decoding caused an additional loss of detected transcripts. Comparing a nonbarcoded osmFISH dataset in mouse somatosensory cortex^2^ with EEL for the same genes showed that the complete end-to-end EEL protocol achieved a 13.2% median per-gene detection efficiency for ready-to-use IDT oPools probe or 2.6% with probes amplified from a Twist Oligo Pool (Supplementary Fig. 2d). One key to RNA capture efficiency was the addition of poly(d-lysine) to the surface, which enhanced the transfer efficiency 60-fold compared to capture by oligo(dT) alone (Supplementary Fig. 2a,b). Compared to the popular spatial transcriptomics platform Visium by 10x Genomics, EEL captured five times more RNA for the overlapping 440 genes when considering only the capture spots of Visium, with a much higher spatial resolution. This increased to a tenfold difference if the total tissue area is considered (Supplementary Fig. 2d,e).

Because the capture surface was also the electrode, we were concerned that electrolysis of water at the anode would lead to a local drop in pH that would interfere with capture by neutralizing or reversing the charge of the RNA molecule. To verify that pH did not drop, we directly measured surface pH by sandwiching particles doped with the pH-sensitive fluorophore pHrodo between the slide and tissue section, which demonstrated that the pH was maintained during electrophoresis (Supplementary Fig. 3).

### Lateral diffusion

Electricity reduced the observed lateral diffusion of the captured RNA on both a large and single-cell scale so that the original tissue organization was preserved by the tissue blot (Supplementary Fig. 2f,g). The distinct cellular pattern of RNA reflecting the sparse localization of cell bodies in the mouse brain was also better preserved when electricity was used (Fig. 2a and Supplementary Fig. 2h). To quantify the preservation of spatial structure, we used Ripley’s L function^24^ that measures deviation from homogeneity. We found a stronger peak for the typical cell size (~10 μm) for conditions that included electricity—as compared with those that did not—indicating that electrophoresis better preserved the original spatial distribution (Supplementary Fig. 2c).

As an independent and more direct estimate of the extent of lateral diffusion, we exploited the sparse distribution of cortical interneurons that express neuropeptides at high levels. We identified individual cells expressing *Vip* (encoding vasoactive intestinal peptide) and measured the distance from the cell centroid to nearby *Vip* transcripts. For EEL data, 50% of the *Vip* transcripts were located within 7.5 µm, compared to 4.4 μm in tissue using smFISH (Fig. 2b). Thus, the lateral diffusion under EEL conditions was smaller than the diameter of one cell on average.

### Barcoding and image acquisition

Capturing RNA on a surface speeds up imaging by reducing or eliminating the *z*-stack and improves the signal-to-noise ratio (SNR) by diminishing background noise normally caused by tissue autofluorescence and scattering (EEL SNR, 49.7 ± 13.2; tissue smFISH SNR, 23.3 ± 11.7; Supplementary Fig. 2i). Together with improvements to the microscope setup, these factors increased the imaging throughput 40-fold compared to osmFISH^2^, reducing the image acquisition time to 76 s mm^−^^2^, compared to 51 min mm^−^^2^ for osmFISH. Additionally, EEL encodes up to 448 genes per channel in 16 rounds, compared to a single gene per channel and round in osmFISH, resulting in another 28-fold increase in throughput. Furthermore, because the RNA is naked on the surface and the reagents and probes do not need to diffuse through a dense tissue sample, the stripping, labeling and stringency wash cycle is reduced to 65 min in comparison to 4–8 hours in tissue.

Altogether, these improvements meant that a complete 448-gene EEL experiment covering 1 cm^2^ of tissue could be completed in 58 hours, many orders of magnitude faster than osmFISH. The EEL protocol was also nearly fully automated, leaving only 4 hours of hands-on processing time.

All datasets reported here were collected with one color channel, but the number of genes can simply be scaled by adding more channels, which is time-efficient because it only requires an additional exposure but no other additional steps. As proof of principle, we demonstrated this by measuring 883 genes in a human glioblastoma sample using two sets of encoding probes corresponding to detection probes carrying Alexa-647 and Cy3 dyes, which resulted in the detection of 883 genes over 87 mm^2^ in 71 hours (Supplementary Fig. 1g).

### Data analysis

smFISH-based spatial transcriptomics methods generate image datasets with the size of several terabytes. To efficiently process these data, we developed pysmFISH, an open-source distributed computing pipeline that automatically performs background filtering, RNA detection, alignment and decoding of barcoded as well as sequential smFISH experiments^25^.

Furthermore, to facilitate the interactive exploration of the resulting point clouds that can contain many millions of molecules per tissue section, we developed FISHscale, which is a Python-based three-dimensional (3D) visualization and analysis tool for single or multiple large-scale point-based spatial transcriptomic datasets^26^. FISHscale leverages graphics processing unit acceleration to enable dynamic zooming, panning and 3D rotation at high frame rates even with tens of millions of dots.

### Regions and borders

**Fig. 3:**
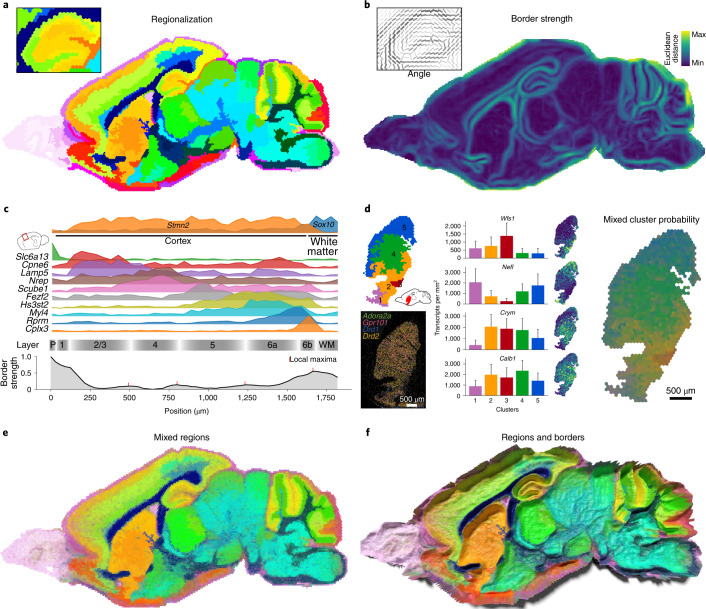
Data-driven anatomical regions and borders. **a**, Clustering of hexagonally binned expression data results in distinct anatomical regions. **b**, Local transcriptional heterogeneity can be quantified to obtain border strength and angle. **c**, Transcript densities for selected genes show layer-restricted expression between the pia and white matter, where interfaces between domains are marked by local maxima in border strength. P, pia; WM, white matter. **d**, The striatum has no clear subregions but contains spatial gradients of certain genes. Mixing region label colors by probability of cluster identity displays these gradients better than discrete colors. Bar plots show mean expression ± s.d. for 2,163 bins. **e**, Mixed cluster colors for the full sample show gradients as well as discrete borders. **f**, Mixed region colors rendered with border strength as height to show the difference between sharp borders and gradients.

Highly multiplexed spatial transcriptome datasets enable the automatic generation of atlases of complex tissues^2,27,28^. We binned RNA spots in a hexagonal grid and performed principal-component analysis (PCA) that could be clustered to generate a regionalized map of the tissue that recapitulated known anatomical structures in the mouse brain (Fig. 3a and Supplementary Fig. 4a–d).

To complement the regionalization, we introduced a border sharpness metric by calculating the local directional dissimilarity as a measure of the strength and orientation of the potential borders (Fig. 3b and Supplementary Fig. 4e,f). We observed the strongest transcriptional difference between gray and white matter, between the cell-dense and cell-sparse regions of the hippocampus and cerebellum and surrounding the striatum and thalamus (Fig. 3b). Weaker, but clear, borders were observed between the layers of the cortex, where genes showed varying layer specificity (Fig. 3b,c and Supplementary Fig. 4f).

In contrast, the striatum contained no clear subregions but instead showed multiple gradients^29^ resulting in arbitrary splits with a clustering approach (Fig. 3d; ref. ^28^). To model such gradients, we trained a classifier on the cluster labels, and then mixed the original region colors based on the class probability of each cluster label (Fig. 3d). This way of visualization preserved highly distinct regions that showed sharp borders, as well as gradients, and could be jointly visualized with border strength (Fig. 3e,f).

### Single-cell resolution

**Fig. 4:**
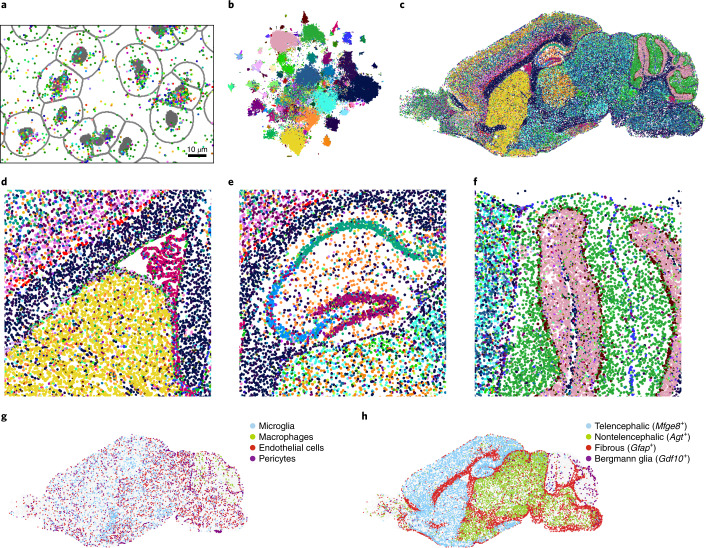
Single-cell analysis of EEL data. **a**, RNA spots assigned to cells inside the expanded segmentation masks of aligned nuclei (gray borders). **b**, *t*-SNE of single-cell profiles where colors indicate the 187 clusters. **c**, Spatial cell type map where every dot is a single cell colored by cluster identity as in **b**. **d**–**f**, Magnified views of **c** showing the ventricle including the rostral migratory stream (**d**), the hippocampus (**e**) and the cerebellum (**f**). **g**, Same as **c** but showing only microglia, macrophages, endothelial cells and pericytes. **h**, Astrocyte types showing distinct spatial distributions.

Assigning expressed RNA molecules to cells enables analysis of cell types and cell states in their spatial context, but it requires segmentation of cell bodies^7,30,31^. To generate single-cell expression profiles from EEL data, images of propidium iodide-stained nuclei taken at low magnification before tissue digestion were segmented with Cellpose^32^, expanded and registered to the RNA signal with the help of fiducial markers so that molecules could be assigned to cells (Fig. 4a and Supplementary Fig. 5a,b; Methods). After quality control, this resulted in an expression matrix of 127,591 single cells and 440 genes, with a total of 5,369,992 transcripts assigned to cells (61% of all molecules detected in this tissue section).

Compared to single-cell RNA-seq of the mouse brain^1^, the EEL dataset had about half the number of molecules, median 40 versus 87, and genes, median 5 versus 11, per cell for 435 genes measured in both datasets (Supplementary Fig. 5c,d). We found good agreement between the two technologies when correlating mean gene expression in the abundant oligodendrocytes (*r* = 0.87) or looking at genes that should, or should not, be expressed in the same cells (Supplementary Fig. 5c,e,f). The most notable difference was that there was slightly more inter-cell-type contamination in EEL, likely due to diffusion of RNA in processes that get assigned to nearby cells. Next, we applied a standard single-cell clustering pipeline (Methods) to construct a spatial map of cell types in the mouse brain, resulting in 187 distinct clusters representing major cell types (Fig. 4b–f). Examining these clusters, we found both abundant cell types with little spatial structure such as microglia and endothelial cells and highly spatially structured neurons and glia (Fig. 4g,h). For example, several distinct types of astrocytes occupied largely nonoverlapping domains (Fig. 4h)—olfactory bulb, the rest of the telencephalon, cerebellum (Bergmann glia) and the nontelencephalic brain. The *Gfap*^+^ fibrous astrocyte subtype was found in white matter and covered most of the brain surface (glia limitans) with the exception of the cerebellum. Cortical layer-specific excitatory and inhibitory neurons were observed, as well as distinct hippocampal neurons of the CA1, CA3, dentate gyrus and molecular layer interneurons (Fig. 4e and Supplementary Fig. 5g). Several clusters corresponded to migrating neuroblasts of the rostral migratory stream into the olfactory bulb and of the dentate gyrus subgranular zone (Fig. 4d,e and Supplementary Fig. 5g).

These results demonstrate the power of EEL to reveal the cell type composition of the mouse brain from a single experiment.

### Mouse brain atlas

**Fig. 5:**
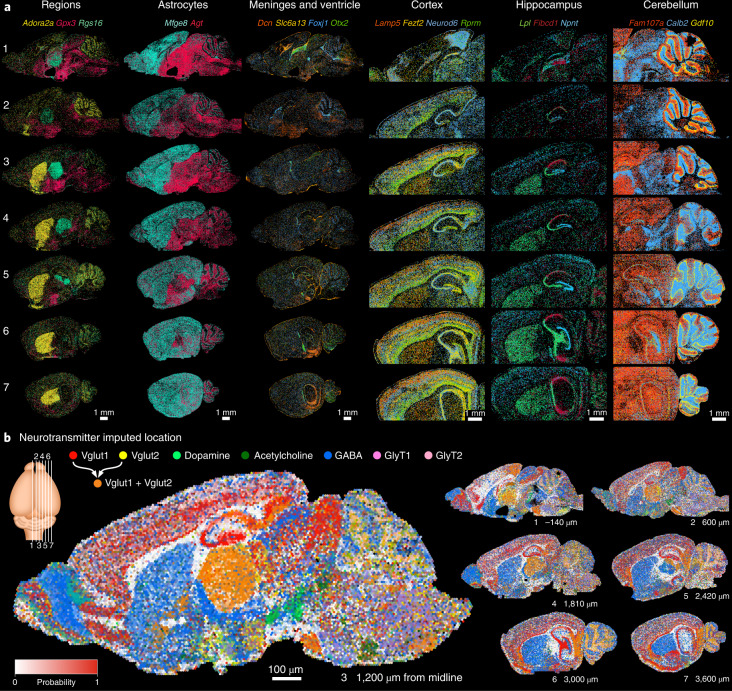
Mouse brain atlas of seven sagittal sections. **a**, Raw RNA signals for selected markers to highlight various anatomical structures. From left to right: markers for striatum (*Adora2a*), hypothalamus (*Gpx3*) and thalamus (*Rgs16*); *Mfge8* labels telencephalic astrocytes, whereas *Agt* labels nontelencephalic astrocytes; markers for meninges (*Dcn*, *Slc6a13*), ependymal (*Foxj1*) and choroid plexus epithelium (*Otx2*); layers of the cortex; hippocampal CA1 (*Fibcd1*), CA3 (*Lpl*) and dentate gyrus (*Npnt*); cerebellar Bergman glia cell body (*Gdf10*), processes (*Fam107a*) and granule cells (*Calb2*). **b**, Probabilities of neurotransmitter location by imputing single-cell RNA-seq data with the spatial mouse atlas show neurotransmitter domains. Intensity reflects the probability of neurotransmitter identity, and colors are proportionally mixed where neurotransmitters overlap.

Next, to demonstrate the robustness and scalability of EEL, we generated an atlas of the mouse brain by measuring the expression patterns of 168 genes in seven sagittal sections starting at the midline and moving laterally with a spacing of ~600 μm, detecting a total of 17,151,357 molecules (Supplementary Fig. 6). We observed region-specific gene expression of various structures, which all showed clear correspondence between consecutive sections (Fig. 5a). This enabled the regionalization of individual sections and linking of the resulting regions in 3D (Supplementary Fig. 7). Interestingly, our data showed in great detail the sharp border between telencephalic and nontelencephalic astrocytes, marked by *Mfge8* and *Agt*, respectively^1^ (Fig. 5a). Having access to multiple sagittal sections confirmed that the border closely followed the telencephalon–diencephalon divide, further reinforcing the notion that these astrocyte types are developmentally specified.

High multiplexing not only facilitates the study of many regions and cell types in a single experiment but also can be used to spatially embed single-cell RNA-seq data. We used a generalized version of Tangram^33^, named Bonefight^34^, to align our previously published single-cell census of the mouse brain^1^ with the spatial mouse atlas, resulting in a putative anatomical location for all cell types, largely in agreement with expected locations (Supplementary Fig. 8a). Once cell types have been aligned, their properties can be transferred to the spatial domain. For example, we generated spatial maps of neurotransmitter usage by summing the spatial probabilities of cell types that share a specific neurotransmitter and projecting them spatially (Fig. 5b and Supplementary Fig. 10b). Overlaying all neurotransmitters in the same section showed the regional preference of one, or sometimes multiple, neurotransmitters. For example, *Vglut1* and *Vglut2* were both present in the thalamus, subiculum and pontine gray (Fig. 5b, orange).

### Human brain atlas

The study of human brain samples by spatial methods has been limited both by the size of the human brain and by the presence of lipofuscin, an age-related accumulation of highly autofluorescent lipid-containing lysosomal residue that is mostly found in neurons. The presence of lipofuscin has precluded study of the human brain and other human tissues in the past using osmFISH because the strong autofluorescence interfered with the weak fluorescence signal from individual mRNA molecules. In contrast, we found that the EEL protocol eliminated most of the lipofuscin and tissue autofluorescence, thus enabling the study of human samples (Supplementary Fig. 9).

**Fig. 6:**
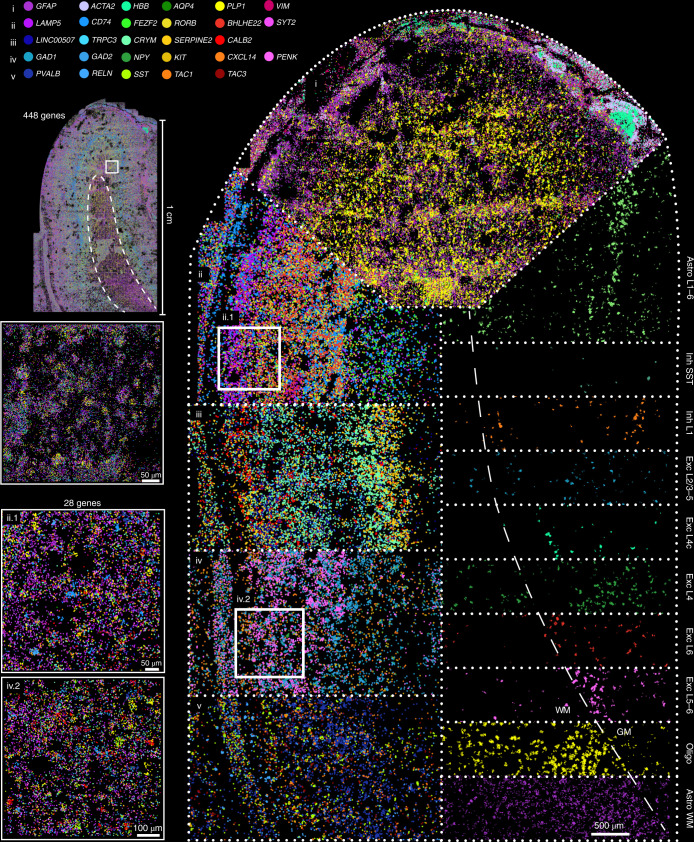
EEL results on adult human visual cortex. Highlighting the RNA spots of 28 genes in the primary visual cortex to show the following: (i) markers for nonneuronal cell types, (ii) superficial layer neuronal markers, (iii) deep-layer neuronal markers and (iv, v) inhibitory neuron markers. On the right side, GraphSAGE molecule embedding clusters show cell types spatially organized in anatomical compartments, such as layers (L), white matter (W.M.) and gray matter (G.M.).

We applied EEL on a 0.75 cm^2^ section of the human primary visual cortex and measured the expression of 445 genes (Fig. 6). Individual genes allowed us to identify region-specific expression patterns repeated along the entire cortical structure: glial and other nonneuronal cell types (Fig. 6i), cortical superficial and deep-layer-specific expression (Fig. 6ii,iii), and spatial distribution of inhibitory cell types (Fig. 6iv,v).

To explore the spatial structure of the human visual cortex, we trained an unsupervised graph neural network constructed on RNA molecule neighborhoods (as proposed in ref. ^35^). We found that molecule embeddings corresponded to spatially and molecularly distinct domains, and we observed that some of these domains corresponded to known cell types of the human cortex, such as excitatory and inhibitory neurons, astrocytes and mature oligodendrocytes (Fig. 6, right). The human visual cortex differs from other cortical areas by the characteristic band of Gennari^36^, which contains axonal tracts carrying visual input from the thalamus, which we could identify along layer 4 by both the spatial location and molecular profile in our data (Fig. 6, layer 4c).

These findings demonstrate that EEL can be successfully applied to human adult brain tissue, as the high level of multiplexing and spatial resolution allowed us to characterize the distinct molecular profiles of cells in the cortex that correspond to cell types or states (Supplementary Fig. 10).

## Discussion

Here we have described a spatial transcriptome profiling method that is capable of high multiplexing, high spatial throughput and high resolution, thus addressing the difficulties in elucidating the spatial distributions of large numbers of cell types within complex tissues and enabling the study of entire sections of mouse brain in just two days of imaging. Additionally, by reducing the detrimental effect of lipofuscin on imaging, EEL enables the investigation of human samples at meaningful scale.

EEL builds on concepts from both microscopy- and sequencing-based spatial transcriptomic methods. Similar to sequencing-based methods^9–15^, we transferred the RNA to a surface, although EEL introduced an active RNA transfer step instead of relying on passive diffusion. We found that electrophoresis better preserved the spatial tissue organization on a cellular scale. Similar to microscopy-based methods, we then detected mRNA molecules in situ using targeted probes^37^.

The main advantage of sequencing-based methods is that they can capture and sequence the full transcriptome. In contrast, EEL—like most microscopy-based techniques—relies on panels of selected probes, albeit with a flexible design that potentially is scalable to thousands of genes. For example, with five color channels, EEL could detect more than 2,000 genes, similar to the typical number of variable genes found in single-cell RNA-seq experiments. With additional imaging cycles and modified barcode schemes, EEL could be scaled further, as has been previously demonstrated for surface-based RNA detection with smFISH (for example, 10,212 genes with RNA SPOTs^37^).

Furthermore, EEL is an order of magnitude less expensive than sequencing-based methods at $600 per experiment (~$0.005 per cell). Additionally, EEL has the advantage that the capture surface is continuous so that there are no gaps between capture features. The diffraction-limited imaging resolution of 200–400 nm enables single-cell transcript assignment through cell segmentation, resulting in a cell-by-gene data matrix.

As a trade-off for high spatial throughput, EEL showed a lower sensitivity compared to tissue-based smFISH methods. However, the reduced optical crowding enabled multiplexing of more genes. Nevertheless, the data quality was close to that of typical single-cell RNA-seq data and allowed for the spatial analysis of both transcriptionally defined regions and their borders, as well as construction of atlases of cell types at the single-cell level. With future improvements to RNA stability and barcode detection, EEL’s sensitivity can be further improved.

EEL makes it possible to analyze whole mouse organs, as shown here for the mouse brain. However, even with greatly increased imaging and chemistry speed, further improvements would be needed to be able to image whole human organs. One factor limiting imaging speed in our current setup was a slight curvature of the capture slide caused by the flow cell design, which we compensated for by imaging a small *z*-stack (Supplementary Fig. 1f). With a flat flow cell, the imaging speed would increase from 76 to 9.3 s mm^−^^2^, so that a 10 cm^2^ sample would be completed in 61 hours. This is roughly the same time as for a 1 cm^2^ sample with the current flow cell and would thus enable EEL to scale to the size of many important human brain structures such as the midbrain, hindbrain and cortical lobes.

Early gene expression atlases (for example, the Allen Brain Atlas^38^) have served as immensely valuable resources for the wider research community. However, enormous resources and complex organization were required to generate such datasets, with the result that they could not be applied to many individuals, diseased tissues or genetic animal models. With the development of highly multiplexed spatial transcriptomics—scalable to large tissue areas and with rapid and robust automation—it now becomes possible to generate bespoke atlases for specific research questions. EEL enables the study of complex tissue specimens at scale and facilitates the study of the molecular organization of the human brain.

## Methods

### Protocol

The full step-by-step protocol is available online^39^ at https://www.protocols.io/view/eel-fish-t92er8e.

### Sample collection

Animal handling and tissue collection methods followed the guidelines and recommendations of local animal protection legislation and were approved by the local committee for ethical experiments on laboratory animals (Stockholms Norra Djurförsöksetiska nämnd, Sweden, N 68/14). Two wild-type CD1 female mice postnatal day 41 were used for the controls, mouse atlas and mouse 448-gene experiments. Mice were transcardially perfused with ice-cold oxygenated artificial cerebrospinal fluid solution. Brains were collected, submerged in Tissue-Tek optimum cutting temperature (OCT Sakura) and snap frozen in a slush of isopentane (Sigma) and dry ice, before storage at −80 °C.

Human brain tissue from a 50-year-old male was performed after obtaining permission from the decedent’s next-of-kin as previously described in ref. ^40^, in accordance with the provisions of the US Uniform Anatomical Gift Act of 2006 described in the California Health and Safety Code section 7,150 (effective 1 January 2008) and other applicable state and federal laws and regulations, and with ethical approval from the Swedish Ethical Review Authority (2019-03054 for human adult brain, 2020-02096 for human glioblastoma). Human serological screening for infectious disease (HIV, hepatitis B and hepatitis C) was conducted using donor blood samples and only considered if it was negative for all three tests. Human cortex V1C was sliced, submerged in Tissue-Tek OCT (Sakura) and snap frozen in a slush of isopentane (Sigma) and dry ice before storing it at −80 °C.

Human colon and breast cancer samples were obtained with the patient’s permission and with ethical approval from the Swedish Ethical Review Authority (2018/1518-31 and 2019-01379 for colon, 2016/957-31 and 2017/742-32 for breast cancer). Samples were frozen as described above.

### Capture slide preparation

ITO coverslips (24 × 60 mm #1.5 thickness, Diamond Coatings) with a surface resistivity of 30–60 Ω per square were cleaned by three successive washes of 20 min in a beaker glass filled with acetone (Sigma), isopropanol (Sigma) and dH_2_O (Thermo) placed in a Ney ULTRAsonik 28X sonicator set to maximum power. Slides were stored in dH_2_O and used between 5 and 30 d after production. To functionalize the surface, the slides were placed in an Epridia E103 rack (Fisher Scientific), dried with nitrogen gas and submerged in a 2% (vol/vol) solution of (3-glycidyloxypropyl)trimethoxysilane in acetone for 2 hours under a nitrogen atmosphere. Coverslips were rinsed once with acetone and dried with nitrogen gas. Then, the oligo(dT) mixture consisting of 10 μM 5′ amine-modified oligo(dT)_60_ (/5AmMC6/UUUUGACTCGTTTTTTTTTTTTTTTTTTTTTTTTTTTTTTT/iSuper-dT/TTTTTTTTTTTTTTTTTTTTTTTTTTT/iSuper-dT/TT, IDT) in 1× Schott Spotting Solution was prepared. The ITO-coated side of the coverslip was identified using a multimeter, and 40 μl of the oligo(dT) mixture was placed on the center and covered with a 24 × 24 mm plastic Hybrisilp (Grace Biolabs). The oligo(dT) mixture was let to react with the epoxy groups for 1 hour at 25 °C. Afterward, the coated slide was washed five times with 2× sodium sodium-citrate buffer (SSC, Sigma) followed by two washes with dH_2_O. Remaining epoxy groups were blocked for 30 min at room temperature with a 0.1% (wt/vol) solution of poly(d-lysine) (molecular weight, 70,000–150,000 (Sigma)) in dH_2_O, followed by three washes with dH_2_O. Europium-doped beads with a diameter of 0.2 and 1 μm (Thermo; 1 μm custom produced) were each diluted 1:333 in dH_2_O and deposited on the surface by placing a 100-μl drop on the coated area for 3 min. The bead mixture was pipetted off and slides were dried in air.

Coated capture slides can be stored for at least 2 d under a nitrogen atmosphere. Cryosections of 10 μm were cut and captured on the coated area and then dried for a few minutes and stored at −80 °C until use. Frozen slides with tissue sections can be stored for a few months before proceeding to the transfer step.

### Probes

Direct labeled smFISH probes for *Malat1*, *Actb*, *Tuba1a*, *ACTB* and *CALM1* were obtained from Biosearch Technologies. Detection probes for barcoded EEL experiments were obtained from IDT and consist of a 5′-conjugated Alexa Fluor 647 dye, followed by a thiol linker (/iThioMC6-D/) and then the 20-nucleotide-long detection sequence (Supplementary Table).

The RNA-binding sequence of the encoding probe design was based on previously reported methods^18,41^ and implemented in the software package Oligopy^23^ (for a similar tool, see PaintSHOP in ref. ^42^). Each probe consisted of a 26- to 32-nucleotide-long sequence, reverse complementary of the target RNA sequence, and one or two overhanging tails containing the six detection sequences of 20 nucleotides each, to which the detection probes can bind. These six sequences were added to the RNA-binding sequence in a random order to reduce the potential steric hindrance effect on signal intensity when they accumulate over sequential rounds of hybridization. Furthermore, readout tails were separated by 2 nucleotides (alternating TT, AT, TA and AA).

RNA-binding regions were selected to have a Gibbs free energy (Δ*G*) at 37 °C of −28 kcal mol^−1^ or less, G+C content of 40–60% and a maximum Δ*G* at 37 °C for hairpin or homodimer formation of −9.0 kcal mol^−1^. Then, probe specificity was analyzed using BLAST, and probes with more than 60% identity to five or more off-target RNA species were dropped. Finally, sets of a maximum of 28 probes that would tile with a minimum gap of 2 bp on the RNA were retrieved, and genes that did not reach a minimum of ten probes were dropped.

To prevent optical crowding, we optimized the distribution of genes over the available barcodes using prior knowledge from single-cell RNA-seq data. Whenever possible, the genes expressed in the same cell type were not labeled in the same decoding cycle.

Encoding probes were ordered either from IDT as oPools (mouse atlas experiment) at ready-to-use concentrations or as Twist Bioscience Oligo Pools for the 448-gene mouse and human experiments.

For the IDT oPool probe sets, the fraction of full-length oligonucleotides was expected to be only ~40% for the 186-nt-long probes, because of truncation during chemical DNA synthesis. Placing two tails both 5′ and 3′ to the RNA-binding sequence (as introduced by multiplexed error-robust FISH in ref. ^18^) would potentially result in truncated probes retaining RNA-binding ability but lacking some of the readout sequences. To avoid the issue, the entire RNA-binding sequence was placed on the 5′ side of the oligonucleotide as one tail, so that only full-length probes could bind the RNA.

For higher-complexity probe sets, Twist Bioscience Oligo Pools were used and amplified using a previously published protocol^43^ with the following adaptations. We performed an initial PCR amplification to generate an intermediate stock and used it as the template for the subsequent experiment, not to exhaust the original probe pool. Then, the probes were amplified again by PCR, followed by in vitro transcription, after which the RNA was purified using the Zymo Oligo Clean and Concentrator. Reverse transcription was performed using a 5′-modified forward primer with an amine group so that the encoding probe could be fixed by paraformaldehyde (PFA) for better signal stability. Finally, RNA was degraded by alkaline hydrolysis and single-stranded DNA (ssDNA) probes were purified. Probe sequences are available in Supplementary Table.

### EEL

Washes were generally performed by pipetting 200 μl of solution onto the sample or, in the cases where a flow cell was used, the internal volume of the flow cell (~400 μl) was replaced at least twice for one wash.

Slides with tissue were thawed, and two reference crosses were drawn on the bottom of the coverslip flanking the tissue sample with a solvent-resistant Moist Mark Plus pen (Cancer Diagnostics Inc.; Fig. 1a). Nuclei were labeled for 5 min with a 1 μg ml^−^^1^ solution of propidium iodide (Sigma) in 2× SSC (Sigma) for mouse 448-gene and human experiments or 1 mg ml^−1^ Hoechst (Sigma) for the mouse atlas experiments, followed by five washes with 2× SSC. The sample was covered with a 24 × 32 mm coverslip spaced by a parafilm gasket, and an overview image of the sample was taken using a ×10 objective. The area of the sample was traced on the overview image and used to generate field of view (FOV) positions for the ×40 and ×60 objectives to cover the entire sample. Then, the nuclei and europium beads were imaged at ×40 magnification and the FOV positions for the ×60 objective were saved together with the relative locations of the reference crosses for later alignment.

Afterward, the tissue was permeabilized for 5 min with 0.1% (vol/vol) Triton X-100 (Sigma), and 10 mM dithiothreitol (DTT, Sigma) in 1× Tris-borate-EDTA (TBE, Thermo) and washed five times with 1× TBE. An electrical wire was mounted on the ITO surface with a conductive copper foil tape (m.nu). The cathode was an uncoated ITO coverslip. The slide was mounted in the EEL holder (Supplementary Fig. 1b), and two 1.5-mm-thick polydimethylsiloxane (Sylgard 184 Dow Corning) spacers were placed on either side of the sample and covered with the cathode slide. The electrophoresis buffer containing 10 mM DTT and 1 M urea (Sigma) in 1× TBE was injected with a gel-loading pipette tip, after which the wires were connected to an RND 320-KA3005D laboratory power supply or a Keithley 2450 sourcemeter, and a potential of 1.5 V (10 V cm^−1^) was applied for 20 min. However, a 1.5-V battery also works. To ensure hybridization, the sample was subsequently incubated for 5 min with a high-salt-concentration buffer 6× SSC (this step was not yet implemented for the mouse atlas experiments). The sample was then washed twice with 2× SSC and digested by three incubations (10 min for mouse and 5 min for human) with digestion buffer containing 1% (wt/vol) SDS (Sigma), 20 mM Tris HCl (Thermo), 2,000 U ml^−1^ Superase (Thermo) and proteinase K (1 U ml^−1^ for mouse and 0.5 U ml^−1^ for human) at 30 °C. This was followed by three washes of 5 min with warm 5% (wt/vol) SDS in 2× SSC at 30 °C and five washes with 2× SSC at room temperature. For the human breast cancer and colon experiments, the digestion was started with a 5-min wash of warm 5% SDS in 2× SSC at 30 °C, followed by two 5-min digestions with 0.5 U ml^−1^ proteinase K in digestion buffer followed by two warm washes with 5% SDS in 2× SSC.

For nonbarcoded experiments, the captured RNA was detected with directly labeled smFISH probes (Biosearch Tech) diluted to 250 nM per probe in hybridization mix containing 10% (vol/vol) deionized formamide (Ambion), 0.1 g ml^−^^1^ dextran sulfate (molecular weight > 500,000, Sigma), 1 mg ml^−1^ *Escherichia coli* tRNA (Roche), 2 mM ribonucleoside vanadyl complexes (RVC, Sigma) and 200 μg ml^−1^ BSA (Sigma) in 2× SSC for at least 30 min at 38.5 °C. Unbound probes were washed away with three washes of 10 min with 20% formamide in 2× SSC at 38.5 °C and five washes with 2× SSC. The sample was subsequently mounted on a microscope slide with Prolong Glass Antifade mounting medium (Thermo).

For barcoded experiments, the RNA was fixed on the surface for 10 min with 4% PFA (Sigma) in 1× PBS (Thermo), followed by five washes with 2× SSC. The appropriate amount of encoding probes was dried using a SpeedVac depending on the probe production’s final concentration as measured by Qubit (Thermo) and resuspended in 20 μl of hybridization mix with 30% formamide. The mix was then carefully pipetted on the sample, covered with a plastic Hybrislip, placed in a petri dish humidified with 2× SSC and incubated for at least 24 hours at 38.5 °C. Afterward, the Hybrislip was carefully removed by first adding some 2× SSC to the edge until it was absorbed under the Hybrislip, creating space between the slide and Hybrislip.

The slide was then mounted in a flow cell (Rebus Biosystems) and placed in the ROBOFISH fluidic system^44,45^, which automatically performed all subsequent steps. The sample was flushed with 2× SSC and washed four times for 15 min with 30% formamide in 2× SSC at 47 °C, followed by four washes with 2× SSC. The encoding probes were then fixed for 10 min with 10% PFA (Sigma) in 1× PBS and washed with 2× SSC. Fluorescent detection probes were dispensed to the sample at a concentration of 50 nM in 10% formamide hybridization mix and hybridized for 10 min at 37 °C, followed by three washes of 3 min with 20% formamide in 2× SSC and four washes with 2× SSC. Imaging buffer containing 2 mM 6-hydroxy-2,5,7,8-tetramethylchroman-2-carboxylic acid (Trolox, Sigma), 5 mM 3,4-dihydroxybenzoic acid (DBA, Sigma) and 20 nM protocatechuate 3,4-dioxygenase from *Pseudomonas* sp. (PCD, Sigma) was then dispensed to the flow cell. Imaging was triggered by the ROBOFISH system and performed on a Nikon Ti2 microscope at 20 °C^46^. After imaging, the sample was washed four times with 2× SSC, and fluorophores were cleaved off by reducing the thiol bond with 50 mM TCEP (Sigma) in 2× SSC during two washes of 10 min at 22 °C. This was followed by ten washes with 2× SSC. Then, 15 cycles of hybridization, washing, imaging and stripping were performed to image all 16 bits of the barcode.

Usually, two staggered experiments were run in parallel. While the first experiment was being imaged (2–3 d), the next experiment was started, hence reducing the downtime of the microscope and doubling the speed by which datasets were generated.

### ROBOFISH automated fluidics

The ROBOFISH system is an open-source fully automated fluidics and temperature control platform integrated with imaging. It is designed to dispense arbitrarily small volumes to a flow cell by bridging the dead volume so that costly solutions like probe mixes are not wasted away. It is designed to be flexible, in terms of both components and running protocols. A syringe pump (Tecan Cavro XE 1000 or Cavro XCalibur) with a Y valve is connected to the running buffer (2× SSC) and a reservoir tube. The reservoir tube is connected to two 10-port actuated valves (MX-II IDEX), which are in turn linked to buffer tubes, up to two flow cells and a waste container.

This setup enables the aspiration of the target buffer into the reservoir, after which extra running buffer is aspirated into the syringe pump so that, when dispensed, the extra volume bridges the dead volume between the valve and flow cell (Supplementary Fig. 1d). Between the valve and flow cell, a bubble trap (Elveflow) and liquid degasser (Degasi Biotech) ensure that no air enters the flow cell. The flow cell itself can be either a flow cell designed by Rebus Biosystems, which is temperature controlled by a TC-720 controller (TE Technology), or the FCS2 flow cell from Bioptechs, which can be temperature controlled by either the Solid State Oasis or the Solid State ThermoCube recirculating chillers. The FCS2 flow cell temperature monitoring is implemented with the Yoctopuce Thermistor. Open-source Python drivers are available (TC-720 (ref. ^47^), ThermoCube^47^, Oasis^48^ and MXII^49^).

The ROBOFISH system monitors buffer volumes and notifies the user via text messages (Pushbullet, Pushbullet python API) when buffers need to be replaced or in the unlikely case of system errors or abnormal temperatures in the room. The full protocol is written to log and metadata files to save all information with the image datasets. Full building instructions, code and operating instructions are available online at https://www.protocols.io/view/robofish-construction-bcrciv2w and https://github.com/linnarsson-lab/ROBOFISH.

### Imaging

Imaging was performed on a Nikon Ti2 epifluorescence microscope equipped with a Nikon CFI Plan Apo Lambda ×60 oil immersion objective with a numerical aperture (NA) of 1.4, CFI Plan Apo Lambda ×40 objective with NA 0.95, CFI Plan Apo Lambda with NA 0.45, Nikon motorized stage, Nikon Perfect Focus system, Sona 4.2B-11 back-illuminated sCMOS camera with 11-μm pixels (Andor), Lumencor Spectra light engine (configuration in Table 1) and matching filter sets (Table 2).

**Table 1:** Light source specifications

Spectral name	Bandpass (nm)	Power (mW)	Measured power ×60 objective (kW cm^−^^2^)
Violet	360/28	400	0.81
Cyan	494/20	400	1.26
Green	534/20	400	2.57
Yellow	586/20	400	1.24
Red	631/28	500	2.80
Red 2	690/10	500	1.38
Near-infrared (NIR)	747/11	500	1.63
NIR2	780/10	500	(Outside range)

**Table 2:** Filter cube specifications

Name	Emission	Dichroic	Excitation
Multiband-89403m-dapi-A590-A750	89403m, https://www.chroma.com/products/parts/89403m	89403bs, https://www.chroma.com/products/parts/89403bs	89403X chroma, https://www.chroma.com/products/parts/89403x
ET525/30-A488	ET525/30m, https://www.chroma.com/products/parts/et525-30m	T505lpxr, https://www.chroma.com/products/parts/t505lpxr	None
ET575/40m-A532	ET575/40m, https://www.chroma.com/products/parts/et575-40m	T550lpxr, https://www.chroma.com/products/parts/t550lpxr	None
ET667/30-A647	ET667/30, https://www.chroma.com/products/parts/et667-30m	T647lpxr, https://www.chroma.com/products/parts/t647lpxr	None
ET740/40X-IR700	ET740/40X, https://www.chroma.com/products/parts/et740-40x	ZT670rdc-xxrxt, https://www.chroma.com/products/parts/zt670rdc-xxrxt	None
RET792lp-IR800	FF01-832/37-32, https://www.semrock.com/filterdetails.aspx?id=ff01-832/37-25	RT785rdc, https://www.chroma.com/products/parts/rt785rdc	None

The automated image acquisition protocol was made in Nikon NIS Elements as a custom job (https://github.com/linnarsson-lab/ROBOFISH). Data acquisition is triggered by the ROBOFISH system and 12-bit images of each FOV with a *z*-stack of 17 slices with a 0.3-μm step. Acquisition of a *z*-stack was necessary to compensate for the curvature of the coverslip, caused by the assembly procedure in the flow cell. A typical sagittal mouse brain section was covered by 500–700 FOVs positioned with an 8% overlap. Alexa-647 and Europium were imaged using the ET667/30-A647 filter cube by switching between 631-nm and 360-nm excitation at 100% and 30% power, respectively, and an exposure of 80 ms for both. After imaging completion, the imaging job notified the ROBOFISH system to continue with the fluidics of the next cycle.

### Surface pH measurement

The surface pH during electrophoresis was measured by fluorescent readout of *E. coli* particles doped with pH-sensitive pHrodo Green fluorophore (Thermo). Ten, 200 μl of 1 mg ml^−^^1^ sonicated particles in 1× PBS were deposited on the capture slide after Europium bead deposition (Supplementary Fig. 3a,b). Afterward, a 10-μm mouse brain tissue section was placed on top and permeabilized as described earlier. A series of buffers with varying pH (MES buffer: 4.7, 5.0, 5.5, 6.0, 6.5; SSC: 7.0 and PBS: 7.4) was used to make a fluorescence calibration curve by measuring the mean pixel intensities of the particles at different pH levels (494 nm excitation, 200 ms exposure time using the ×60 objective; Supplementary Fig 3c). Large aggregates of particles were excluded from the analysis.

An additional sample was placed in the electrophoresis holder, and electrophoresis buffer was injected (TBE pH 8.3, 1 M urea, without DTT) and mounted on the microscope. Images of two FOVs were taken every 45 s and after 2.5 min of baseline acquisition, electrophoresis at 1.5 V was performed for 20 min. During this time, the mean measured fluorescence did slightly increase but never in the range of the calibration curve, indicating that the buffer maintains the pH (Supplementary Fig. 3d). In contrast, when higher voltages (4 V and above) were applied, we did observe a drop in pH at the surface as interpolated with the calibration curve (for example, electrophoresis at 6 V in Supplementary Fig. 3e,f). The higher the voltage, the faster the pH drop, and the effect was more pronounced at surfaces covered by the tissue, thus indicating that restricted diffusion augments the surface pH change.

### Image analysis pipeline

We developed a processing pipeline to automatically detect and decode the EEL signal^25^ called pysmFISH. A flow chart of the analysis steps is available on the GitHub page. Briefly, after parsing, filtering and counting, the detected spots are registered between hybridizations using the Europium fiducial beads. The barcodes are then identified using the nearest-neighbor approach. To map the EEL signal (×60 objective) to the nuclei images (×40 objective), we first registered the Europium beads imaged with both objectives by using a point set alignment algorithm based on a nearest-neighbor search, and the resulting transformation (scaling, rotation and shift) was applied to the EEL signal. Nuclei were segmented using Cellpose^32^, and the segmentation masks were expanded by 8 μm without overlapping. Detected signal dots were then assigned to cell labels if they fell inside a segmentation mask making use of a k-d tree algorithm. To process the large amount of data generated by each EEL experiment, the analysis is parallelized using Dask^50^.

### Optical density

Optical density simulations were performed by filling a 10 × 10 μm^2^ area with an increasing number of randomly spaced points and determining how many could be resolved using Abbe’s diffraction limit (*λ*/2NA) as the minimal distance. The simulation was repeated for various wavelengths of light and for objectives with different NAs.

### SNR

SNR was defined as the ratio between the signal strength and the standard deviation of the background pixels surrounding the signal spot (annulus with radii of three and nine pixels). If other signal spots fell in the vicinity of the signal in question, these pixels were excluded from the standard deviation calculation. The SNR was measured on the *Tuba1a* signal on samples processed by EEL and smFISH in tissue of the experiment presented in Fig. 2a. *Tuba1a* was labeled in the same channel as in the barcoded EEL experiments.

### Technique comparison

To compare the detection efficiency of EEL to that of osmFISH and Visium, we selected data of anatomically identical areas using the Allen brain atlas as a guide. The anterior and posterior Visium data for sagittal section number 2 were downloaded from www.10xgenomics.com on 18 March 2022. The Visium sequencing library had a sequencing depth of 73,558 reads per spot and was sequenced to 81% saturation. The osmFISH dataset is available on http://linnarssonlab.org/osmFISH/. The per-gene RNA density was calculated for the somatosensory cortex areas of all EEL samples. The comparison with single-cell RNA-seq was made to a whole mouse brain single-cell dataset in ref. ^1^ available at http://mousebrain.org/adolescent/; http://mousebrain.org/adolescent/ with a mean sequencing depth of 42,629 per cell and was sequenced to 74.4% saturation. For all comparisons, only genes that were measured in both datasets were considered.

### Spatial analysis of mouse samples

Lateral diffusion was investigated by identifying *Vip*^+^ cells in the osmFISH data and the seven mouse atlas experiments through DBSCAN. Their centroid was calculated based on the location of the molecules, after which the probability of finding a molecule in concentric circles around the centroid was calculated and compared.

To facilitate the exploration and analysis of point-based spatial datasets that contain millions of molecules, we developed a Python package called FISHscale (https://github.com/linnarsson-lab/FISHscale), which efficiently handles large datasets by storing them on disk and relying on parallelized processing with Dask^50^. FISHscale can handle multiple datasets for analysis and 3D visualization. The visualization tool is based on Open3D^51^, which enables rapid visualization of millions of molecules.

FISHscale implements a method to regionalize the tissue sample by binning the data in hexagonal bins, performing PCA or Latent Dirichlet Allocation (LDA) and clustering the hexagons using Ward hierarchical clustering making use of the spatial information by using a connectivity matrix for the neighboring hexagonal tiles. The resulting region labels can be ordered by spectral embedding to give transcriptionally similar regions a similar color. If multiple regionalized datasets share anatomical structures between them, these regions can be linked by correlating their mean expression.

To visualize the mixture of region identities, a random forest classifier was trained on the hexagonally binned data with the region labels, and the probability of each region label was obtained by querying the classifier. These probabilities were then multiplied by the red, green and blue (RGB) values of original region colors and summed to display gradients in the form of mixed colors.

Border strength was calculated by placing a grid of points over the sample and selecting all molecules within a 200-μm radius from each point. Each group of selected molecules was then split in half 12 times at different angles. For each angle, we calculated the total number of molecules in each half and measured the Euclidean distance between the counts. A large distance corresponds to a bigger difference in gene expression between the regions separated along a specific angle. This allows us to measure both the strength and the angle of the potential border (Fig. 4b). In the 3D rendered image (Fig. 4f; Blender, https://www.blender.org/), the border strength is visualized as the height of the mixed region colors.

To align the hexagonally binned spatial datasets with a previously published single-cell RNA-seq study of the mouse nervous system^1^, we used a generalized version of Tangram^33^ called Bonefight^34^ to calculate the probability distributions of the location of each of the 199 cell types that were identified in the mouse brain. The neurotransmitter identity of the various cell types was summed to give the probability of the spatial location of each neurotransmitter.

### Spatial analysis of human samples

The human data were segmented using an unsupervised graph-based approach^52^ in which the algorithm enforces that connected nodes should have similar embeddings, whereas randomly sampled pairs of nodes should have dissimilar embeddings. We built a graph neural network of two layers with 24 hidden units, a rectified linear unit activation function between the layers, a pooling aggregation and differentiable group normalization. The graph was built using the Deep Graph Library’s SAGEconv module (https://www.dgl.ai/). Each RNA molecule formed a node in the graph, and any two molecules were connected by an edge if their distance was below 15 μm. During training, mini-batches of 512 nodes were generated, for each center node a neighborhood one hop and two hops away is subsampled (maximum 20 nodes for the first hop and 10 nodes for the second hop), each layer aggregates and updates the information of each node and its sampled neighborhood. After training, an embedding was generated for every molecule, and *k*-means clustering was used to cluster molecules into distinct spatial domains. The genes enriched in each spatial domain were used for annotation.

### Statistics and reproducibility

No statistical tests have been performed. The results in Fig. 1 have been repeated two times with similar results. The results in Fig. 2a have been repeated two times with similar results. The results in Supplementary Fig. 1g have been repeated 25 times with similar results. The results in Supplementary Fig. 2f,g have been repeated two times with similar results. All other experiments have been presented in full. The method as a whole has been repeated at least 85 times by various people on various tissues of mouse and human in health and disease.

### Reporting summary

Further information on research design is available in the Nature Research Reporting Summary linked to this article.

## Online content

Any methods, additional references, Nature Research reporting summaries, source data, extended data, supplementary information, acknowledgements, peer review information; details of author contributions and competing interests; and statements of data and code availability are available at 10.1038/s41587-022-01455-3.

## Supplementary information

Supplementary Information Supplementary Figs. 1–10.

Reporting Summary

Supplementary Table 1 Sequences for all probes used in the EEL manuscript.

## Data Availability

All data are available on http://mousebrain.org/ and https://figshare.com/projects/Scalable_in_situ_single-cell_profiling_by_electrophoretic_capture_of_mRNA_using_EEL_FISH/143616 Supplementary Table 1 lists probes. The EEL FISH protocol is available in ref. ^39^. ROBOFISH building instructions are available in ref. ^45^. Raw EEL FISH images totaling roughly 18 TB are available on request.

## Code availability

Olygopy software to design barcoded smFISH probes is from ref. ^23^. ROBOFISH software to control automated microsfluidics and imaging is from ref. ^44^. pysmFISH image analysis software for multiplexed smFISH is from ref. ^25^. FISHscale spatial analysis software is from ref. ^26^. The TC-720 temperature controller Python driver is from ref. ^53^. The ThermoCube recirculating temperature controller Python driver is from ref. ^47^. The Oasis recirculating temperature controller Python driver is from ref. ^48^. The MXII actuated valve Python driver is from ref. ^49^.
