# Supplementary material for: Scalable in situ single-cell profiling by electrophoretic capture of mRNA using EEL FISH
Source: Nat Biotechnol. 2022 Sep 22;41(2):222–31. doi: 10.1038/s41587-022-01455-3 (PMC9931581; doi:10.1038/s41587-022-01455-3)
Supplement: Supplementary file 1 — Supplementary Figs. 1–10. [file 41587_2022_1455_MOESM1_ESM.pdf]

nature portfolio

https://doi.org/10.1038/s41587-022-01455-3

Supplementary information

Scalable in situ single-cell profiling by electrophoretic capture of mRNA using EEL FISH

In the format provided by the authors and unedited

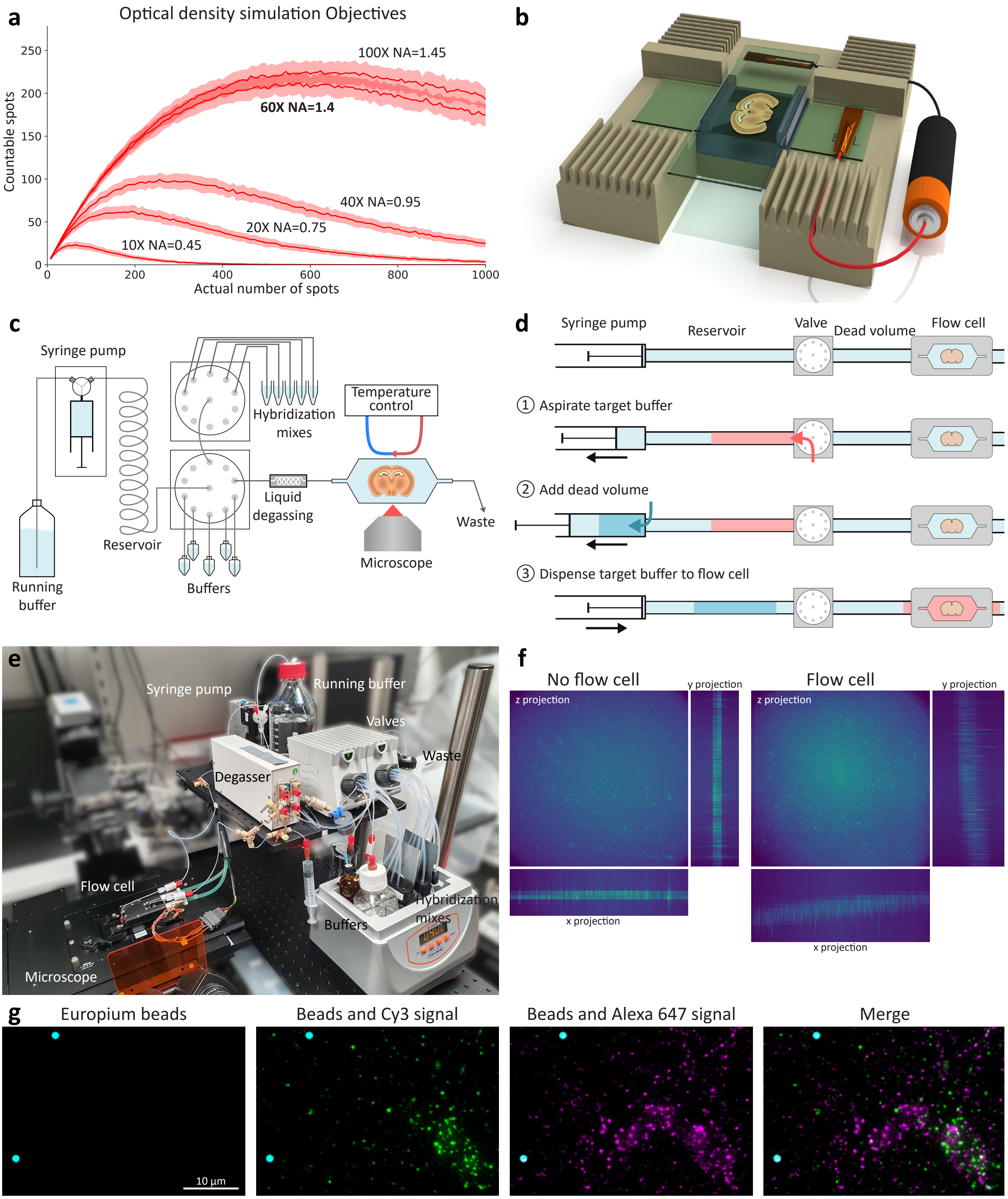

Supplementary Fig. 1 | EEL setup. a, Computer simulation of countable dots for Alexa 647, defined as not overlapping with any other dot within the Abbe diffraction limit, for increasing numbers of dots in a virtual cell for various objective lenses. Mean +/- standard deviation as shaded areas for 100 simulations. **b**, EEL electrophoresis setup. The tissue section on the capture slide is placed on the bottom of the 3D printed holder and connected to the positive pole of a power source. The top electrode is spaced with a silicone strip and connected to the negative pole of the power source. **c**, Simplified schematic of the ROBOFISH fluidic system. **d**, Working principle of the ROBOFISH fluidic system. The target liquid is aspirated into the reservoir. Then the dead volume is aspirated into the syringe. When the full volume is dispensed to the flow cell the target liquid will reach the flow cell, without having to fill the dead volume. **e**, Image of a ROBOFISH system. **f**, x,y and z-projections of a FOV imaged without a flow cell shows RNA on a flat surface, while the flow cell causes a slight curvature in the surface. Z-step is 300nm. **g**, Example of two-color EEL experiment encoding up to 896 (2 x 448) genes.

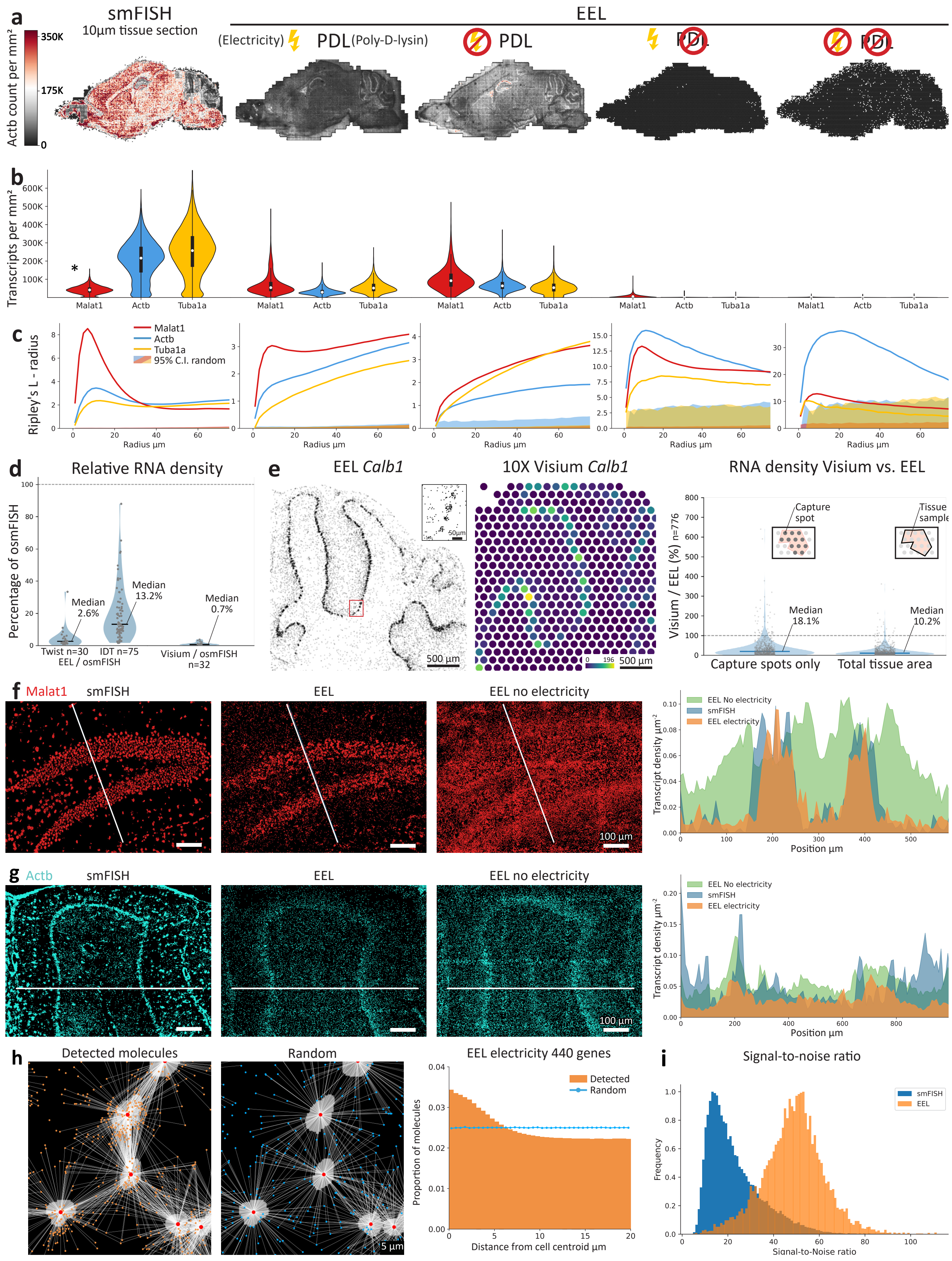

Supplementary Fig. 2 | EEL performance. a, *Actb* molecule counts in adjacent sections for: smFISH in a 10 µm tissue section, EEL with electrophoresis and PDL, EEL without electrophoresis but with PDL, EEL with electrophoresis but without PDL and EEL without electrophoresis and without PDL. Quantified in binned data and normalized to mm^2. **b**, Quantification of 3 genes in the same experiments as in **a**, showing increased RNA capture in EEL conditions with PDL. * *Malat1* is highly expressed, therefore the dots start to overlap, and quantification is likely an underestimate of the real number of molecules. Violin plots show median, 25th and 75th percentile for 14,952, 16,852, 16,289, 14,675 and 14,652 bins respectively from left to right. **c**, Ripley's L score minus

the radius for the same experiments as in **a**. Showing the 95% confidence interval (C.I., solid fill) of randomized data for the same number of molecules, which centre around 0, meaning no deviation from random. In the original situation (smFISH in tissue) the RNA locations of all genes show various degrees of clusterdness with a peak around the average cell size. The addition of electricity in the EEL protocol improves the clusterdness as compared to RNA transfer only by diffusion. **d**, Per gene RNA density comparison of EEL and Visium with tissue smFISH (osmFISH). For EEL a distinction is made between experiments with probes derived from Twist oligo pools (n=30 genes) or IDT oPools (n=75 genes over 5 experiments). For Visium only the area of the capture spots is considered (n=32 genes). **e**, EEL and Visium spatial resolution side-by-side where Visium spots are plotted to scale. Per gene RNA density comparisons between EEL and Visium. For Visium the comparison is made for the area of the capture spots only on the left, or to the total sample area including the space in-between capture spots on the right (n=440). **f**, **g**, *Malat1* and *Actb* transcript localization in dentate gyrus and cerebellum respectively, for smFISH in a 10 µm section, EEL with electrophoresis and EEL without electrophoresis. Density profiles on the right show that EEL with electricity better matches the original tissue structure. In the case of *Malat1* the no-electricity condition shows a very distorted blot. **h**, Measured distances between all detected molecules in the 440 genes sagittal mouse brain section and the nucleus centroid of the cells. Repeating this measurement with randomized data indicated that molecules are more likely to be found close to the centroids, supporting that the RNA transfer matches the sparse cellular architecture of the mouse brain. **i**, Signal-to-noise ratio for in-tissue smFISH and EEL signal where the ratio is defined as the peak spot intensity divided by the standard deviation of the pixels around the spot.

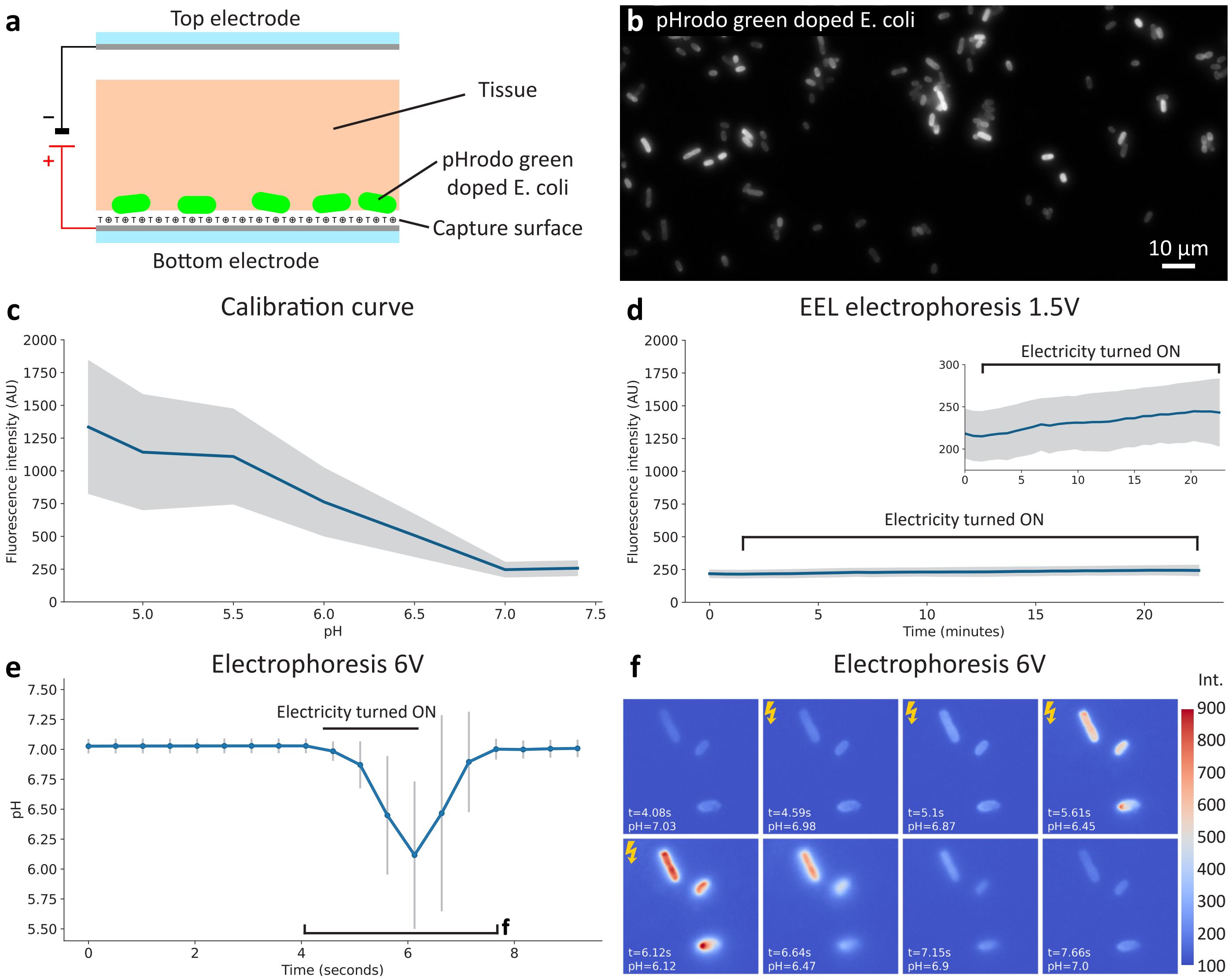

Supplementary Fig. 3 | The EEL electrophoresis step does not lower surface pH. a, Experimental setup with pHrodo green doped *E. coli* sandwiched between the capture slide and the tissue, which is the place where the RNA will be captured, and pH could have an effect. **b**, Image of particles at physiological pH. **c**, Calibration curve by measuring fluorescence intensity at various pH levels. **d**, Fluorescence intensity measured every 45 seconds during a regular 20 minute EEL electrophoresis step at 1.5 Volt. Data shown with the same y-scale as in **c**. A very slight increase was observed (see inset with rescaled y-axis). However, fluorescence intensity stayed below the calibration curve indicating no detrimental drop in pH. **e**, **f**, When performing electrophoresis at higher potentials, the surface pH rapidly drops. Mean intensity values of *E. coli* containing pixels are shown where shaded areas in **c**,**d** and error bars in **e** represent standard deviations for 71,205 pixels in **c**, 11,646 pixels in **d** and 9,658 pixels in **e**.

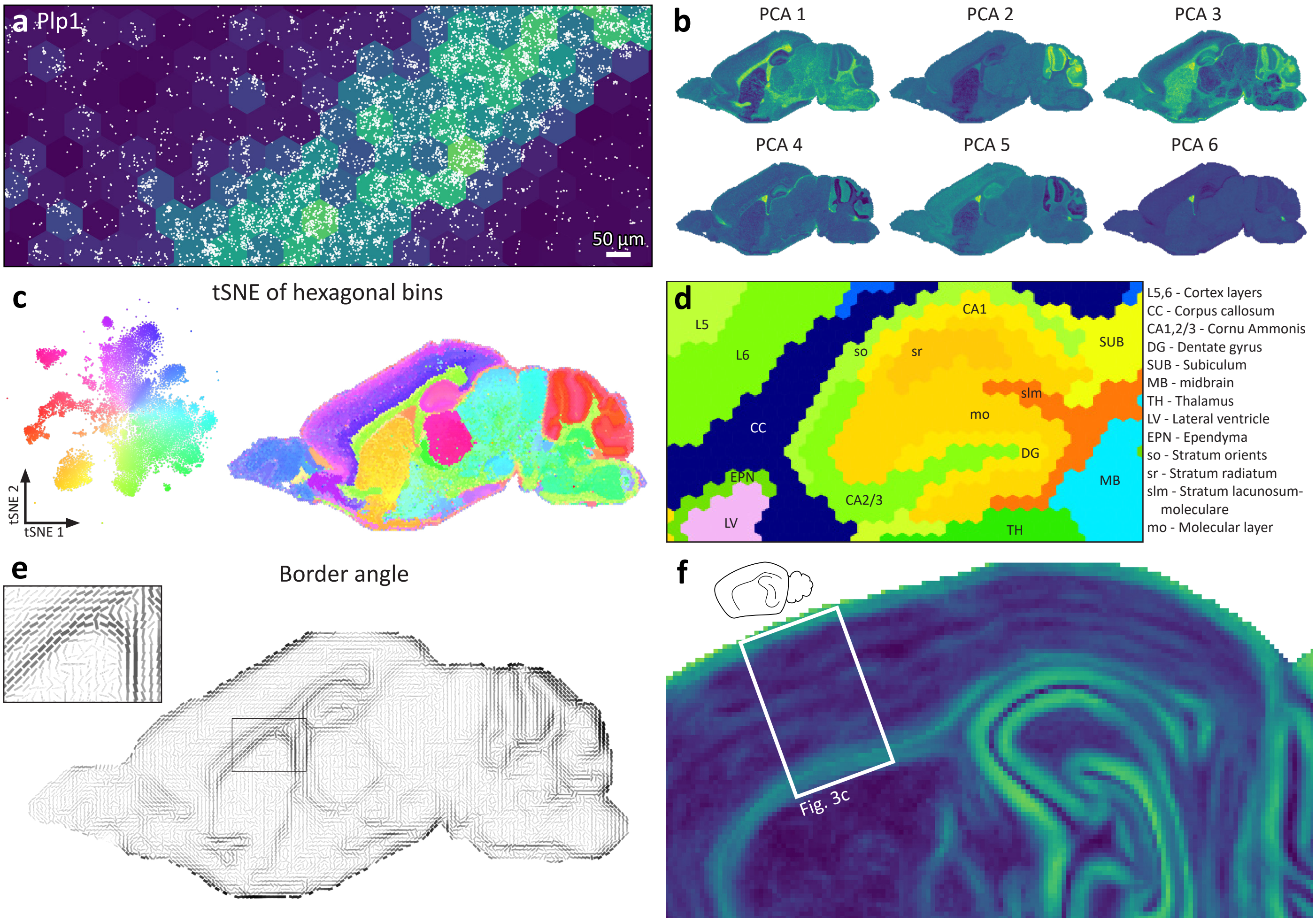

Supplementary Fig. 4 | Data-driven regionalization of mouse brain. a, Hexagonal binning of detected signal, here showing *Plp1* as an example. **b**, First 6 PCA components of hexagonally binned data shows that components capture different anatomical structures. **c**, t-SNE embedding of hexagonal bins also show that anatomical structures are captured by the components. **d**, Detailed view of the regionalization in the hippocampus with labelled anatomical regions. **e**, Angle of the largest transcriptional difference indicating border direction. Line width and darkness correspond to border strength. **f**, Border strength of section 6 of the mouse atlas that is used for Fig. 3c, showing borders in the cortex.

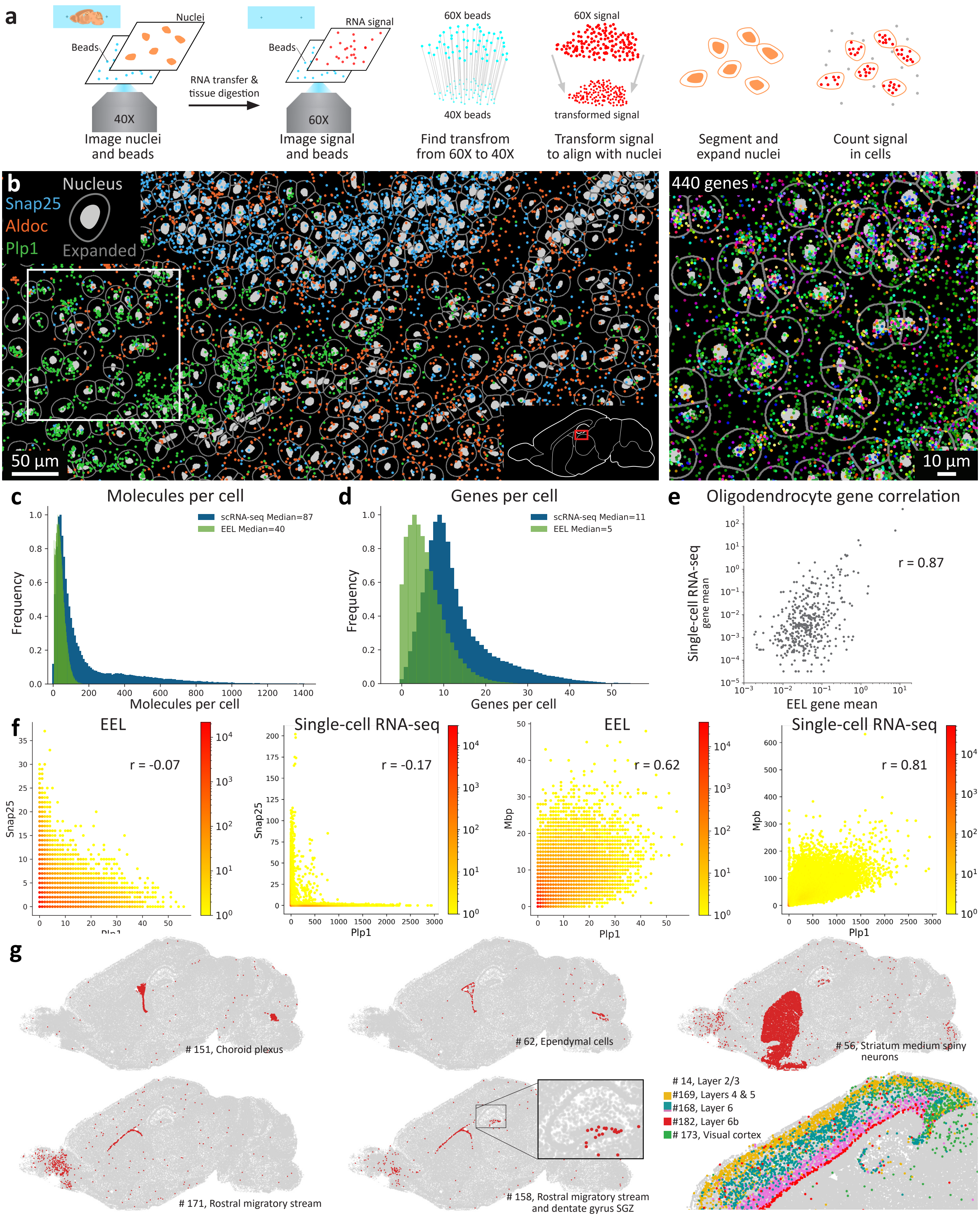

Supplementary Fig. 5 | Single cell segmentation and clusters. a, Pipeline to segment cells. Before the RNA is transferred and the tissue is digested, the nuclei are imaged at 40X magnification along with fiducial beads. These beads are also imaged when imaging the signal at 60X magnification so that they can be used to match the images before and after tissue removal. The signal is transformed to fit the space of the 40X images. Nuclei are segmented and expanded as a proxy for the cell boundary. Then, RNA is counted in the segmented and expanded masks to generate a gene-by-cell matrix. **b**, Example region showing segmentation in white matter, hippocampus and thalamus for 3 genes (top), or all 440 genes (bottom). **c,d**, Comparison of molecules per cell (**c**) and genes per cell (**d**), for EEL single cell profiles with single-cell RNA-seq profiles for the overlapping 435 genes. **e**, Correlation of mean gene expression of 435 genes in oligodendrocytes identified by EEL and single-cell RNA-seq. **f**, Correlation of gene expression of genes that should not be found in the same cell-type (Snap25 and Plp1) or genes that should be in the same cell type (Mbp and Plp1). Density is indicated by color. **g** Spatial locations of EEL cluster examples. Choroid plexus, expressing *Kl*, *Foxj1*, *Aqp1.* Ependy-

mal cells *(Ccdc153, Foxj1, Tmem212).* Striatum medium spiny neurons *(Adora2a, Gpr88, Drd1, Drd2)* Rostral migratory stream *(Dlx1, Meis2, Sox11).* Subventricular zone of the lateral ventricle and subgranular zone of the dentate gyrus *(Sox11, Igfbpl1, Hes5)*; inset confirms the location of dentate gyrus stem cells along the hilus border. Clusters of distinct cortical layers.

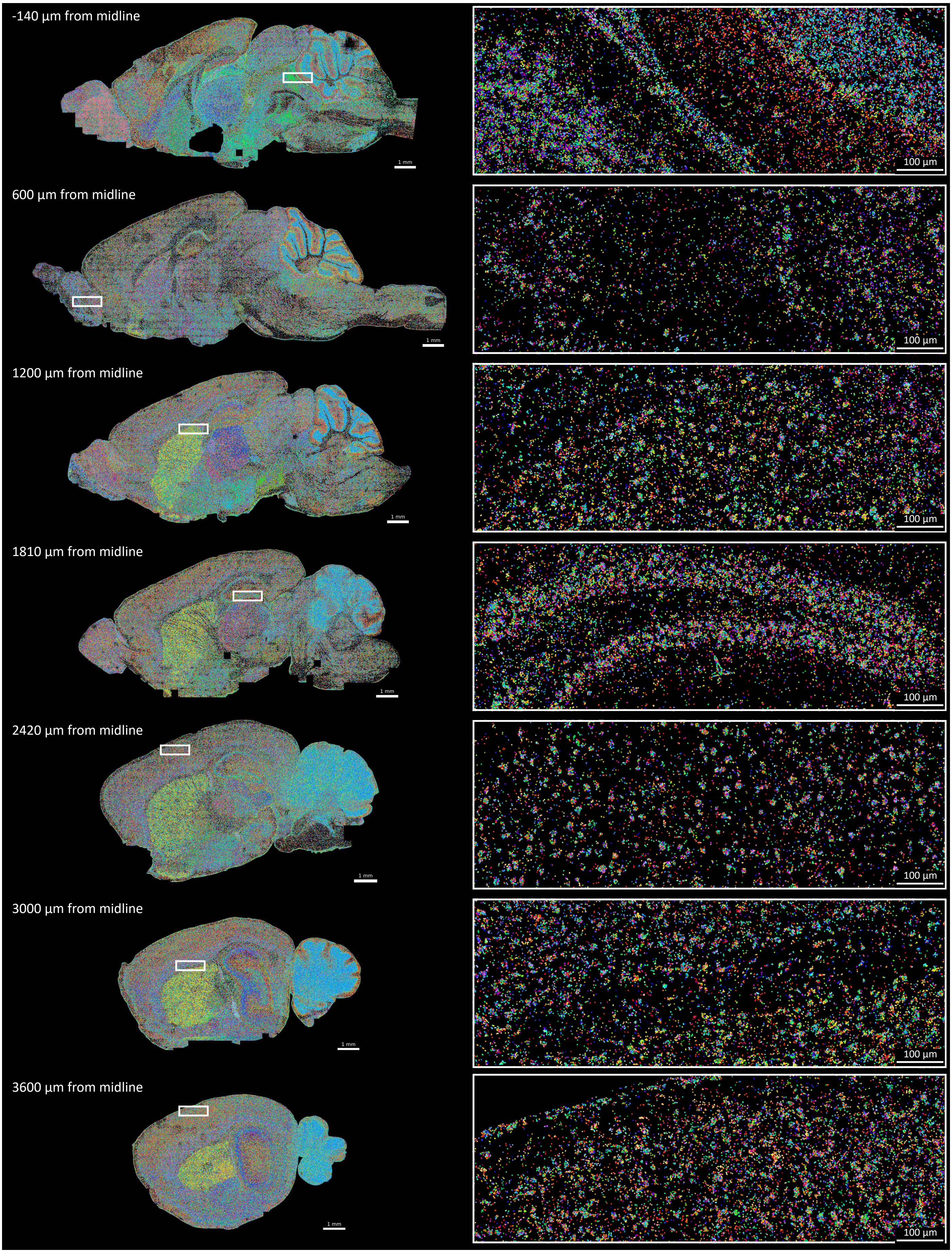

Supplementary Fig. 6 | Raw data of the 7 sections of the sagittal mouse atlas. Colors correspond to one of 168 measured genes.

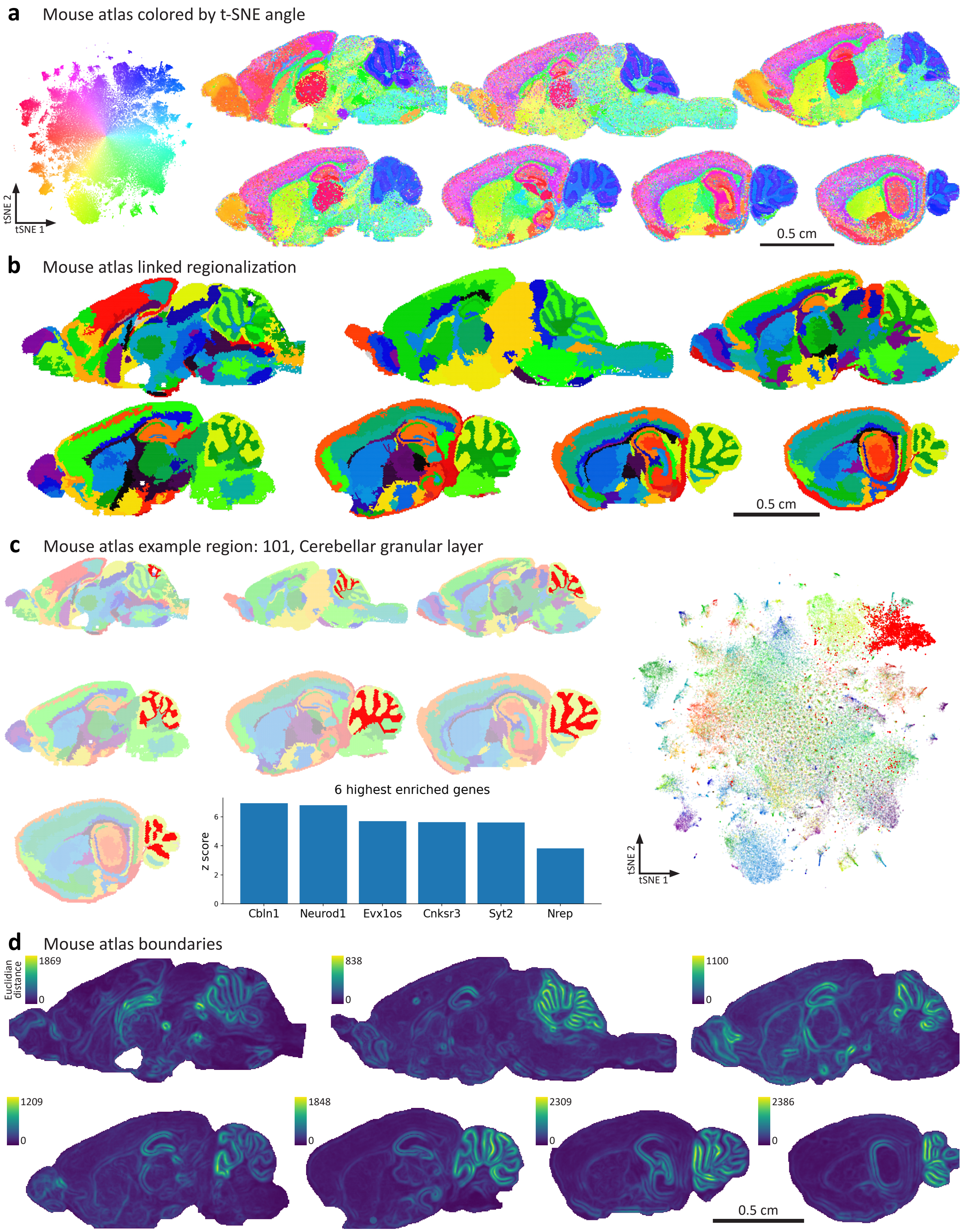

Supplementary Fig. 7 | Mouse atlas regionalization and borders. a, t-SNE run on the combination of all hexagonal bins of the 7 sections, indicating that datasets integrate well as similar anatomical structures from adjacent sections co-localize on the t-SNE without showing obvious batch-to-batch variation. **b**, Regionalized mouse brain atlas where similar regions are linked between adjacent sections. **c**, Example of regions that link between all sections of the mouse atlas. Spatial location and location in the t-SNE is indicated in red. **d**, Boundary strength was measured on all sections and showed matching border locations between the same anatomical structures from adjacent sections.

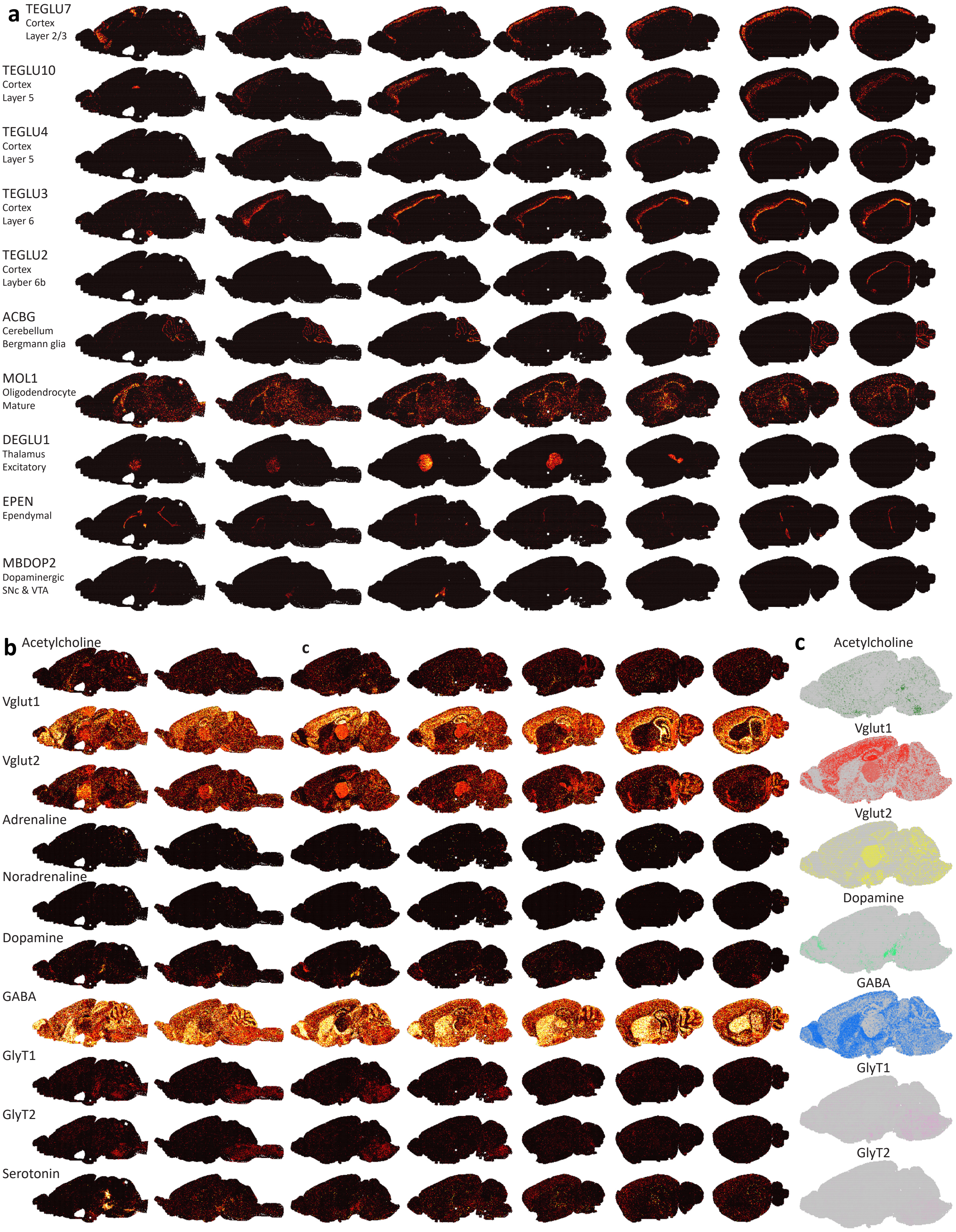

Supplementary Fig. 8 | Integration of the spatial mouse atlas with single-cell RNAseq of the mouse brain. a, Likelihood of the spatial locations of cell types as found by single-cell RNAseq. **b**, Likelihood of spatial location of neurotransmitters in the mouse brain atlas. **c**, Integration of the location of 7 neurotransmitters in the 3rd section by mixing the colors of individual neurotransmitters shows both separated and shared neurotransmitter domains.

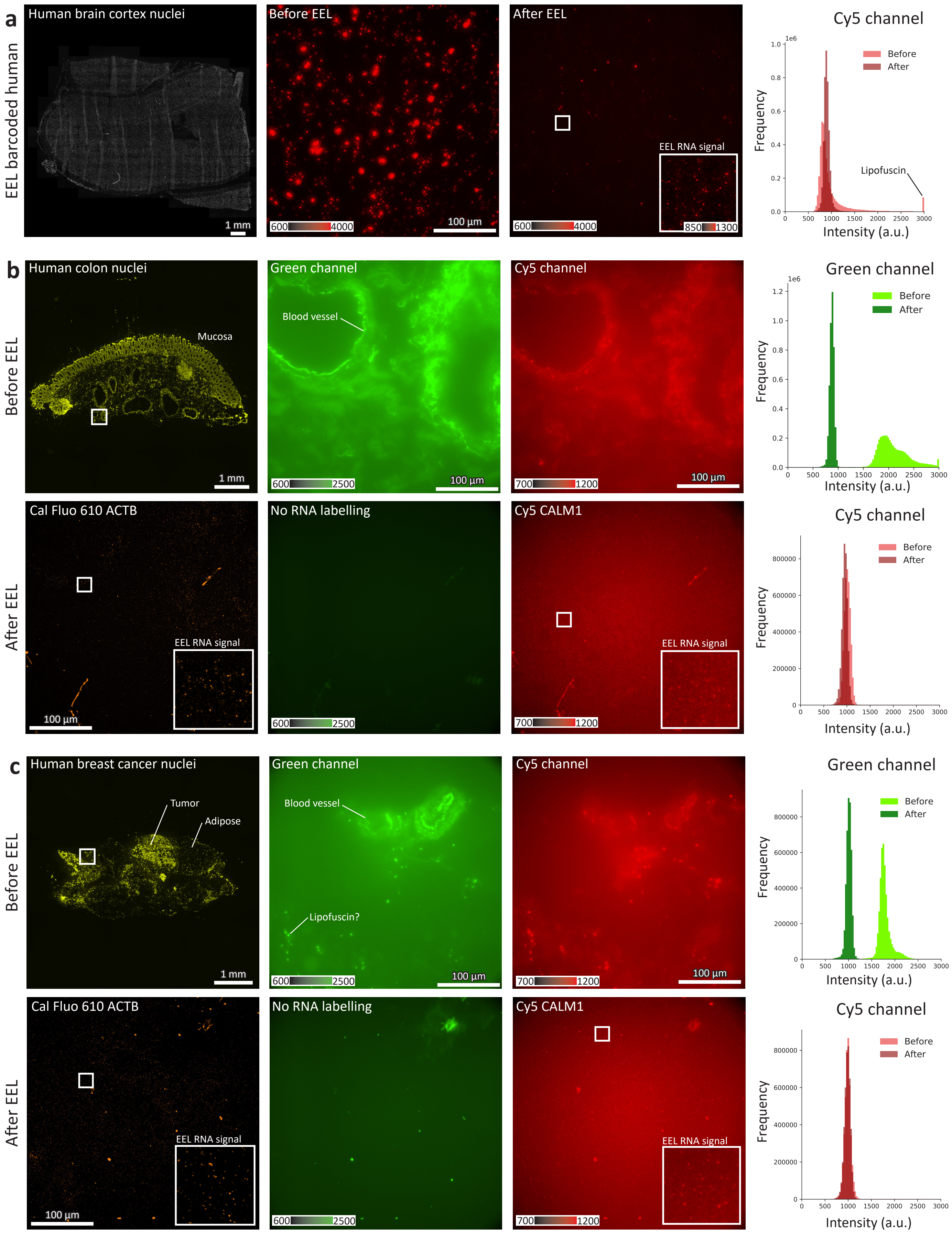

Supplementary Fig. 9 | EEL significantly reduces lipofuscin and tissue autofluorescence. Images of tissue background before and after EEL tissue digestion and RNA labelling. Images before and after were rescaled to the same minimum and maximum, and were taken with the same objective, illumination settings and exposure time. **a**, Human brain cortex. Left panel shows the nuclei of the human adult primary visual cortex of Fig. 6 rotated 90 degrees to the left. Images before and after RNA transfer and tissue digestion show a stark reduction in lipofuscin content. Small dots on the right image correspond to the RNA signal spots (inset), while brighter dots are remaining lipofuscin. **b**, Human colon showed high intensity tissue autofluorescence, which was reduced by EEL digestion so that RNA of ACTB and CALM1 could be detected. **c**, Human breast cancer had both tissue autofluorescence and lipofuscin which was diminished by EEL digestion so that RNA of ACTB and CALM1 could be detected.

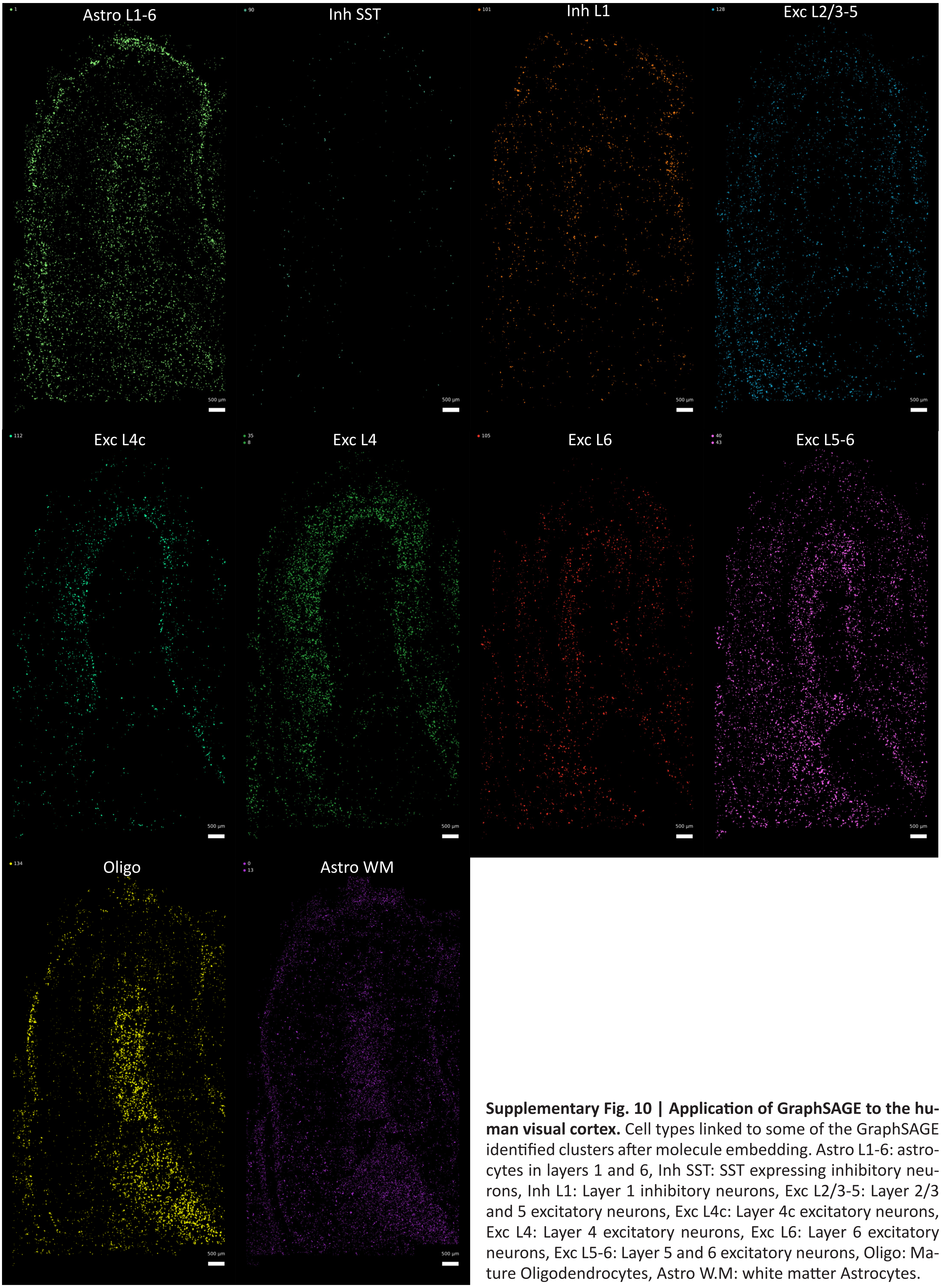

Supplementary Fig. 10 | Application of GraphSAGE to the human visual cortex. Cell types linked to some of the GraphSAGE identified clusters after molecule embedding. Astro L1-6: astrocytes in layers 1 and 6, Inh SST: SST expressing inhibitory neurons, Inh L1: Layer 1 inhibitory neurons, Exc L2/3-5: Layer 2/3 and 5 excitatory neurons, Exc L4c: Layer 4c excitatory neurons, Exc L4: Layer 4 excitatory neurons, Exc L6: Layer 6 excitatory neurons, Exc L5-6: Layer 5 and 6 excitatory neurons, Oligo: Mature Oligodendrocytes, Astro W.M: white matter Astrocytes.
