# Supplementary material for: Scalable in situ single-cell profiling by electrophoretic capture of mRNA using EEL FISH
Source: Nat Biotechnol. 2022 Sep 22;41(2):222–31. doi: 10.1038/s41587-022-01455-3 (PMC9931581; doi:10.1038/s41587-022-01455-3)
Supplement: Supplementary file 2 — Reporting Summary [file 41587_2022_1455_MOESM2_ESM.pdf]

nature portfolio

Corresponding author(s): Lars E. Borm & Sten Linnarsson

Last updated by author(s): Jul 16, 2022

Reporting Summary

Nature Portfolio wishes to improve the reproducibility of the work that we publish. This form provides structure for consistency and transparency in reporting. For further information on Nature Portfolio policies, see our Editorial Policies and the Editorial Policy Checklist.

Statistics

For all statistical analyses, confirm that the following items are present in the figure legend, table legend, main text, or Methods section.

n/a	Confirmed	
☐	☒	The exact sample size (*n*) for each experimental group/condition, given as a discrete number and unit of measurement
☐	☒	A statement on whether measurements were taken from distinct samples or whether the same sample was measured repeatedly
☒	☐	The statistical test(s) used AND whether they are one- or two-sided<br>*Only common tests should be described solely by name; describe more complex techniques in the Methods section.*
☒	☐	A description of all covariates tested
☒	☐	A description of any assumptions or corrections, such as tests of normality and adjustment for multiple comparisons
☒	☐	A full description of the statistical parameters including central tendency (e.g. means) or other basic estimates (e.g. regression coefficient) AND variation (e.g. standard deviation) or associated estimates of uncertainty (e.g. confidence intervals)
☒	☐	For null hypothesis testing, the test statistic (e.g. *F*, *t*, *r*) with confidence intervals, effect sizes, degrees of freedom and *P* value noted<br>*Give P values as exact values whenever suitable.*
☒	☐	For Bayesian analysis, information on the choice of priors and Markov chain Monte Carlo settings
☒	☐	For hierarchical and complex designs, identification of the appropriate level for tests and full reporting of outcomes
☒	☐	Estimates of effect sizes (e.g. Cohen's *d*, Pearson's *r*), indicating how they were calculated

Our web collection on statistics for biologists contains articles on many of the points above.

Software and code

Policy information about availability of computer code

Data collection	Nikon NIS Elements AR version 5.20.02 was used for image acquisition.
Data analysis	Nikon NIS Elements AR version 5.20.02<br>pysmFISH: https://github.com/linnarsson-lab/pysmFISH_auto<br>ROBOFISH: https://github.com/linnarsson-lab/ROBOFISH<br>FISHscale: https://github.com/linnarsson-lab/FISHscale<br>Open3D version 0.14: http://www.open3d.org/<br>Cellpose version 1: https://github.com/mouseland/cellpose

For manuscripts utilizing custom algorithms or software that are central to the research but not yet described in published literature, software must be made available to editors and reviewers. We strongly encourage code deposition in a community repository (e.g. GitHub). See the Nature Portfolio guidelines for submitting code & software for further information.

Data

Policy information about availability of data

All manuscripts must include a data availability statement. This statement should provide the following information, where applicable:

- Accession codes, unique identifiers, or web links for publicly available datasets
- A description of any restrictions on data availability
- For clinical datasets or third party data, please ensure that the statement adheres to our policy

All data available on: http://mousebrain.org/ and https://figshare.com/projects/Scalable_in_situ_single-cell_profiling_by_electrophoretic_capture_of_mRNA_using_EEL_FISH/143616
Supplemental table: probes.
EEL FISH Protocol (Reference 39).
ROBOFISH building instructions (Reference 45).
Raw EEL FISH images totalling roughly 18TB are available upon request.

Human research participants

Policy information about studies involving human research participants and Sex and Gender in Research.

Reporting on sex and gender	The human Brain cortex sample was from a male.
Population characteristics	The donor of the human brain cortex was 50 years old.
Recruitment	Not applicable.
Ethics oversight	Swedish Ethical Review Authority (2019-0305, 2020-02096, 2018/1518-31, 2019-01379, 2016/957-31 and 2017/742-32)

Note that full information on the approval of the study protocol must also be provided in the manuscript.

Field-specific reporting

Please select the one below that is the best fit for your research. If you are not sure, read the appropriate sections before making your selection.

☒ Life sciences ☐ Behavioural & social sciences ☐ Ecological, evolutionary & environmental sciences

For a reference copy of the document with all sections, see nature.com/documents/nr-reporting-summary-flat.pdf

Life sciences study design

All studies must disclose on these points even when the disclosure is negative.

Sample size	The paper describes a new method and does not test a hypothesis, so no sample size estimates were performed. Any references to n numbers are results of data binning, number of genes or number of cells found/included in specific experiments. No statistical tests have been performed on these.
Data exclusions	No data was excluded
Replication	The method was replicated many times as indicated in each figure. For example, Fig 5 shows seven replicates on different sagittal sections of the same mouse, Fig 1 shows a sagittal section from a different mouse and Fig 6 show the method performed on a human section. Supplementary Figure 12 shows additional replicates in human breast cancer and normal colon.
Randomization	Not applicable, no statistical tests have been performed.
Blinding	Not applicable, no statistical tests have been performed.

Reporting for specific materials, systems and methods

We require information from authors about some types of materials, experimental systems and methods used in many studies. Here, indicate whether each material, system or method listed is relevant to your study. If you are not sure if a list item applies to your research, read the appropriate section before selecting a response.

Materials & experimental systems

n/a	Involved in the study
☒	☐ Antibodies
☒	☐ Eukaryotic cell lines
☒	☐ Palaeontology and archaeology
☐	☒ Animals and other organisms
☒	☐ Clinical data
☒	☐ Dual use research of concern

Methods

n/a	Involved in the study
☒	☐ ChIP-seq
☒	☐ Flow cytometry
☒	☐ MRI-based neuroimaging

Animals and other research organisms

Policy information about studies involving animals; ARRIVE guidelines recommended for reporting animal research, and Sex and Gender in Research

Laboratory animals	Two wild-type CD1 female mice postnatal day 41.
Wild animals	Not applicable.
Reporting on sex	Not applicable.
Field-collected samples	Not applicable.
Ethics oversight	Animal handling and tissue harvesting methods followed the guidelines and recommendations of local animal protection legislation, and were approved by the local committee for ethical experiments on laboratory animals (Stockholms Norra djurförsöksetiska nämnd, Sweden, N 68/14).

Note that full information on the approval of the study protocol must also be provided in the manuscript.
